# Time and phenotype-dependent transcriptome analysis in AAV-TGFβ1 and Bleomycin-induced lung fibrosis models

**DOI:** 10.1038/s41598-022-16344-7

**Published:** 2022-07-16

**Authors:** Benjamin Strobel, Holger Klein, Germán Leparc, Birgit E. Stierstorfer, Florian Gantner, Sebastian Kreuz

**Affiliations:** Boehringer Ingelheim Pharma GmbH & Co. KG, Birkendorfer Str. 65, 88397 Biberach an der Riss, Germany

**Keywords:** Preclinical research, Target validation, Transcriptomics, Mouse, Respiratory tract diseases

## Abstract

We have previously established a novel mouse model of lung fibrosis based on Adeno-associated virus (AAV)-mediated pulmonary overexpression of TGFβ1. Here, we provide an in-depth characterization of phenotypic and transcriptomic changes (mRNA and miRNA) in a head-to-head comparison with Bleomycin-induced lung injury over a 4-week disease course. The analyses delineate the temporal state of model-specific and commonly altered pathways, thereby providing detailed insights into the processes underlying disease development. They further guide appropriate model selection as well as interventional study design. Overall, Bleomycin-induced fibrosis resembles a biphasic process of acute inflammation and subsequent transition into fibrosis (with partial resolution), whereas the TGFβ1-driven model is characterized by pronounced and persistent fibrosis with concomitant inflammation and an equally complex disease phenotype as observed upon Bleomycin instillation. Finally, based on an integrative approach combining lung function data, mRNA/miRNA profiles, their correlation and miRNA target predictions, we identify putative drug targets and miRNAs to be explored as therapeutic candidates for fibrotic diseases. Taken together, we provide a comprehensive analysis and rich data resource based on RNA-sequencing, along with a strategy for transcriptome-phenotype coupling. The results will be of value for TGFβ research, drug discovery and biomarker identification in progressive fibrosing interstitial lung diseases.

## Introduction

Progressive fibrosing interstitial lung diseases (PF-ILD) comprise a group of rare disorders characterized by pulmonary fibrosis, lung function decline, respiratory symptoms, and mortality^1^. The concept of PF-ILD arose from the fact that besides idiopathic pulmonary fibrosis—the most common and severe condition among ILDs—a range of additional ILD diagnoses with diverging characteristics but a common manifestation of pulmonary fibrosis is observed in the clinic. These include environmental lung disease, sarcoidosis, idiopathic non-specific interstitial pneumonia (iNSIP), chronic fibrosing hypersensitivity pneumonitis (HP) and connective tissue disease (CTD)-associated ILD.

For IPF, various irritants including smoking, occupational hazards, viral and bacterial infections as well as radiotherapy and chemotherapeutic agents (like, e.g., Bleomycin) have been described as potential risk factors, together with genetic predisposition^2,3^. While genetic factors in other PF-ILDs are largely unknown, the concept of repeated lung injury followed by a self-sustaining fibrotic response that is uncoupled from the initial irritant is hypothesized to be a common feature. Accordingly, pulmonary fibrosis is initiated through repeated alveolar epithelial cell micro-injuries, leading to the recruitment of immune cells and stem/progenitor cells that secrete various pro-inflammatory cytokines, chemokines, and growth factors, thereby triggering expansion and activation of fibroblasts^4^. In contrast to physiological, self-limiting wound healing, continuous deposition of extracellular matrix (ECM) components by activated, contractile myofibroblasts results in progressive lung stiffening and destruction of lung architecture.

Because the most used preclinical lung fibrosis model based on pulmonary Bleomycin instillation only partially reflects aspects of human ILDs, we have recently developed an alternative, complementary mouse model based on Adeno-associated-virus (AAV) 6.2-mediated expression of TGFβ1^5^. In stark contrast to previously used Adenovirus-based models^6,7^ that only induce transient TGFβ1 expression due to antiviral immune responses^8,9^, the AAV-TGFβ model leads to persistent expression of TGFβ and consequently to a progressive worsening of lung fibrosis. Moreover, contrary to the patchy, bronchocentric fibrosis pattern in the Bleomycin model, AAV6.2 specifically targets TGFβ1 expression to bronchial epithelium and alveolar type II (AT2) cells^5^, thereby inducing more homogenous fibrosis, reminiscent of the histological features in non-specific interstitial pneumonia (NSIP)^10^.

In the present work, we thoroughly characterized AAV-TGFβ1 induced pulmonary fibrosis development by simultaneous analysis of phenotypic and bulk RNA-sequencing derived transcriptomic changes (mRNA, miRNA) over a time course of four weeks. By conducting this analysis in direct comparison to Bleomycin-induced fibrosis, we provide a comprehensive insight into the differences and commonalities of these two disease models. Finally, in a transcriptome-phenotype coupling approach based on correlation of longitudinal gene expression changes and lung function measurements, we identify proteins and miRNAs that represent attractive starting points as novel anti-fibrotic drug targets.

## Results

### Phenotypic fibrosis development following AAV-TGFβ1 versus Bleomycin administration

We have previously shown that AAV6.2-mediated TGFβ1 expression in the lung of mice induces fibrosis in a dose-dependent fashion^5,11^. To study the time course of fibrosis development in a direct comparison with Bleomycin-induced lung injury, the most commonly used model of lung fibrosis, C57Bl/6 mice received either 2.5E + 11 vector genome-containing particles (vg) of single-stranded AAV6.2-CMV-TGFβ1 (AAV-TGFβ1) or 1 mg/kg Bleomycin by intratracheal (i.t.) administration. Control mice either received AAVs containing non-coding “stuffer”-DNA (AAV-stuffer) or NaCl. Disease development was followed over day 3, 7, 14, 21 and 28 after administration by several phenotypic and molecular biological analyses, which also included isolation of RNA from lung homogenates for total and small RNA-seq.

**Figure 1:**
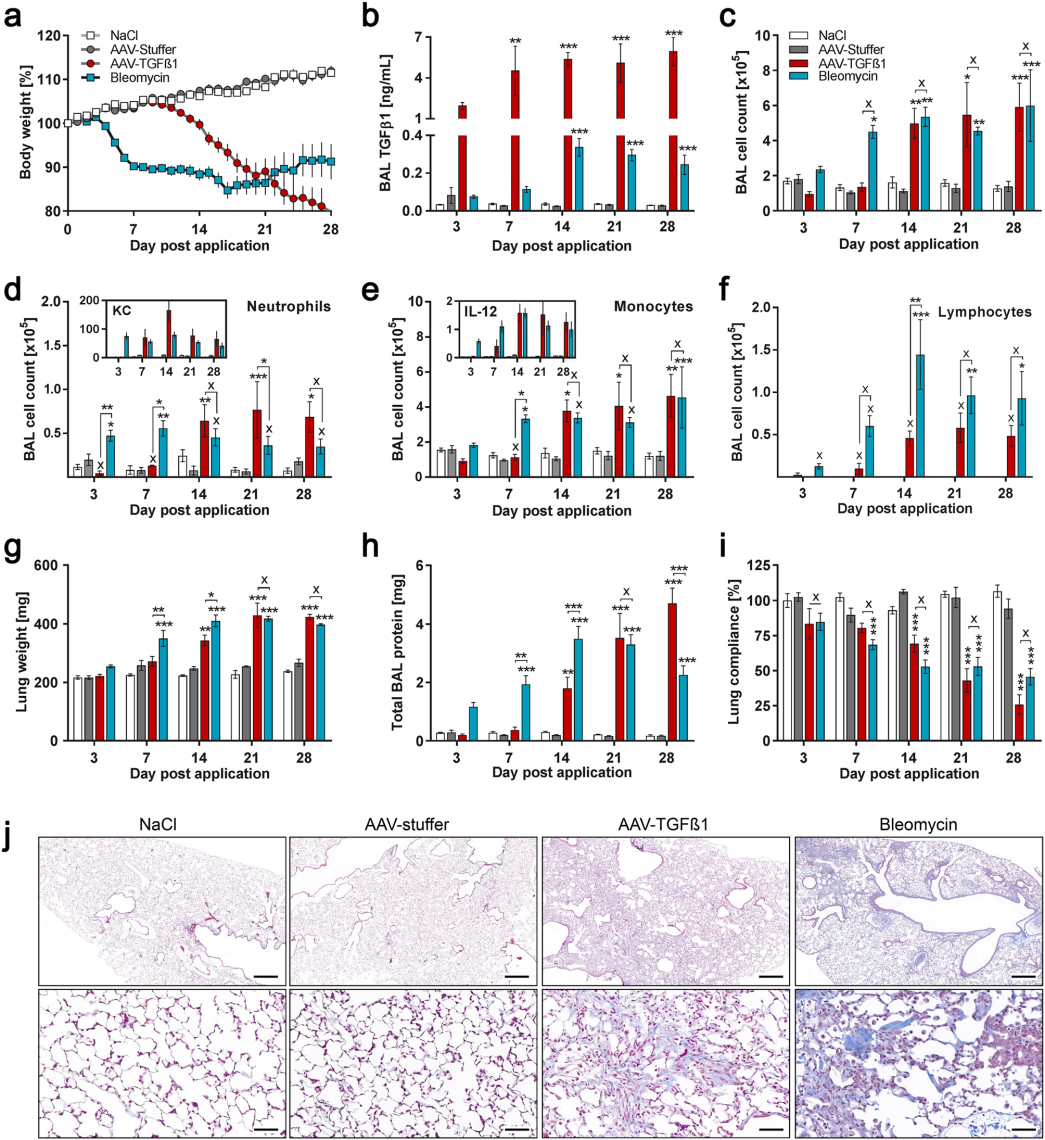
Phenotypic characterization of AAV-TGFβ1 and Bleomycin induced fibrosis. Mice either received 2.5 × 10^11^ vg AAV-TGFβ1 (n = 5 animals per time point), 2.5 × 10^11^ vg AAV-stuffer (n = 5), 1 mg/kg Bleomycin (n = 8) or NaCl (n = 6) via intratracheal administration. Three, 7, 14, 21 and 28 days after administration, lung function was measured prior to sampling of bronchoalveolar lavage (BAL) fluid and extraction of total lung tissue RNA. (**a**) Relative body weight over time. (**b**) Total BAL TGFβ1 protein levels. (**c**) Total BAL cell count and differential counts for (**d**) Neutrophils, (**e**) Monocytes and (**f**) Lymphocytes. Insets show BAL protein levels of KC (= CXCL1) and IL-12. (**g**) Wet lung weight. (**h**) Total BAL protein. (**i**) Lung function measurement. (**j**) Representative Masson-trichrome stained lung tissue sections obtained at day 21 after start of the experiment. Low magnification (upper panel, scale bar = 500 µm) and high magnification (lower panel, scale bar = 50 µm) micrographs are shown. Mean ± SD. *p < 0.05, **p < 0.01, ***p < 0.001 in comparison to respective controls (AAV-TGFβ1 vs. AAV-stuffer, Bleomycin vs. NaCl) or as indicated.

Body weight measurement showed that mice that had received Bleomycin started to lose weight three days after administration, whereas AAV-TGFβ1 mice showed a similar loss in body weight, however, only starting from approximately one week after vector application (Fig. 1a). Notably, while Bleomycin-treated animals started to regain weight from about 18 days post treatment onwards, AAV-TGFβ1-treated animals continuously lost weight, reaching 20% loss at four weeks after starting the experiment. Interestingly, despite the finding that as early as day three, bronchoalveolar lavage (BAL) TGFβ1 levels in the AAV model strongly exceeded the amount of endogenous TGFβ1 produced in the Bleomycin model (Fig. 1b), phenotypic changes were only observed in the Bleomycin model. This clearly illustrates that acute injury and inflammation drive disease onset in the Bleomycin model, whereas in the absence of injury, prolonged TGFβ1 expression is required to disrupt tissue homeostasis and drive fibrosis. This notion is also supported by the finding that neutrophils were distinctly increased at day three in the Bleomycin model (Fig. 1d), followed by an increase in macrophages and lymphocytes from day seven onwards (Fig. 1e,f). While immune cell influx was expected upon acute injury, it was interesting to observe that a similar immune cell influx was also observed in the TGFβ1 model, however, only from day 14 onwards (Fig. 1c–f). This delay in innate immune response clearly demonstrates that it was not targeted towards the viral vector (in line with our previous observation^5^) but rather a consequence of prolonged TGFβ1 activity. The increase in neutrophils and macrophages correlated well with the BAL levels of the neutrophil chemoattractant CXCL1 and the dendritic cell and macrophage product IL-12, respectively, in both models (Insets in Fig. 1d,e).

The different kinetics of phenotypic changes in the models (i.e., acute onset in the Bleomycin model versus onset at day 7 in the TGFβ1 model) was also evident in the increase in lung weight (Fig. 1g), which is an indicator of inflammation and increased extracellular matrix (ECM) production. Moreover, an acute increase in total BAL protein, indicative of disrupted barrier function (i.e., epithelial and endothelial leakage), was observed upon Bleomycin instillation, whereas in the AAV model similar effects were observed only at later time points (Fig. 1h). As a consequence of fibrosis and the corresponding increase in tissue rigidity, a decrease in lung function was observed in both models with the time shift previously observed for all other readouts in the AAV model (Fig. 1i). However, despite the different kinetics in disease onset, a remarkably similar phenotype was observed at later time points, particularly day 21, in both models. Finally, histological analyses confirmed strong fibrosis development in both, Bleomycin- and AAV-TGFβ1-treated animals, with a rather patchy distribution in the Bleomycin model as opposed to a more even distribution of fibrotic areas in the AAV model (Fig. 1j), congruent with our previous observation^5^. This is also mirrored by a slightly higher Ashcroft score at day 21 in the Bleomycin model (Suppl. Fig. 1). These differences are explainable by the different modes of disease induction, i.e., direct and bronchocentric injury with focal inflammation by Bleomycin, and bronchial epithelium and AT2 cell-specific secretion and spreading of TGFβ1 in the AAV model.

Interestingly, despite the strong and persistent overexpression of TGFβ1 in the AAV model, pharmacological intervention by applying the TGFBR1/ALK5 antagonist SB-525334^12^ one week after AAV administration protected the mice from body weight loss, reduced inflammation, and fully blocked fibrosis development (Suppl. Fig. 2). This proof-of-concept experiment also demonstrates suitability of the model for pharmacological intervention studies and profiling of drug candidates.

### Time-resolved transcriptional profiling of fibrosis development following AAV-TGFβ1 or Bleomycin administration

**Figure 2:**
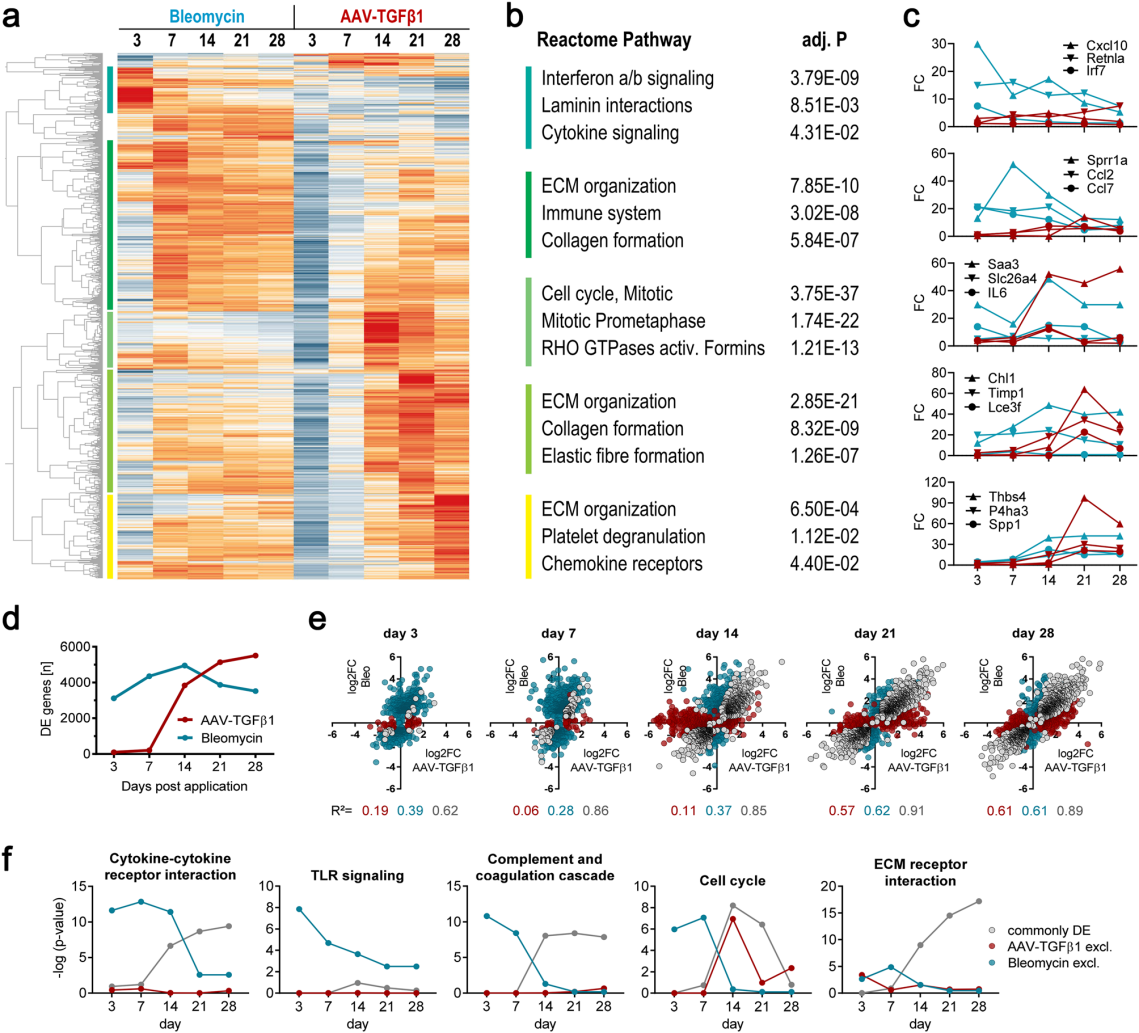
Longitudinal assessment of transcriptomic changes in AAV-TGFβ1 and Bleomycin induced fibrosis. Total RNA was extracted from the mice treated as described in Fig. 1 and applied to RNA-sequencing. (**a**) unsupervised hierarchical clustering (Z-scored FPKMs) of all mRNAs showing differential expression (log_2_FC ≥ 0.6, q ≤ 0.05) at ≥ two contrasts across all time points and models (n = 6010). The genes within the color-coded clusters were applied to (**b**) Reactome pathway enrichment analysis. Cluster 1, n = 148 genes; cluster 2, n = 552; cluster 3, n = 186, cluster 4, n = 403 and cluster 5, n = 275. (**c**) Expression profiles of the top 3 differentially expressed genes in each cluster. (**d**) Total number of differentially expressed genes over time for both models. (**e**) Correlation plots for genes showing differential expression either exclusively in one model or commonly in both models, as defined by expression cutoffs (log_2_FC = 0.6). The coefficient of determination (R^2^) is show for each set of genes under the graphs. Legend as in (**f**). (**f**) Significance of enrichment (*p*-value) for selected KEGG pathways over time, obtained and plotted for the gene sets defined in (**e**). TLR = toll-like receptor. ECM = extracellular matrix. Heatmap created with TIBCO Spotfire Analyst 10.3.2.

To characterize the transcriptional changes associated with fibrosis disease development, mRNA and miRNA were purified from the lungs of AAV-TGFβ1- or Bleomycin-treated animals at day 3, 7, 14, 21 and 28 after administration and analyzed by RNA-sequencing. The number of differentially expressed (|log_2_FC|≥ 0.6, adjusted p-value ≤ 0.05) genes over time (Fig. 2d), unsupervised hierarchical clustering (Fig. 2a) and correlation of model-specific or commonly deregulated genes (Fig. 2e) nicely mirrored the chronology of phenotypic disease manifestation, i.e., specific effects early on day 3 in the Bleomycin model, which are absent in the AAV model, as well as greater similarity between the models during the fibrotic phase (day 14–28), particularly on day 21. A list of the top 20 up-regulated genes per time point is available in the supplement (Suppl. Table 1). Moreover, also a very similar induction of collagen genes was observed across models over time, with a faster onset again observed in the Bleomycin model but a tendency towards slightly higher induction of the highly abundant collagens in the AAV model (Suppl. Fig. 3). These data underscore our finding of a similar overall degree of fibrosis development, as also suggested by the phenotypic data in Fig. 1.

To study longitudinal disease events in more detail, we first analyzed gene clusters showing differential expression at different time points during disease development (Fig. 2a). Pathway enrichment analysis of Cluster 1, which largely contained genes acutely and specifically upregulated in the Bleomycin model at day 3, clearly indicated cellular and DNA damage, involving type I interferon and cytokine signaling (Fig. 2b). The three cluster-genes showing strongest upregulation in the Bleomycin model on day 3 were the ones encoding Cxcl10, an interferon-induced macrophage-attracting chemokine, inflammation- and injury-associated Resistin-like alpha (Retnla/Fizz1) and Interferon regulatory factor 7 (Irf7) (Fig. 2c). A big cluster of genes prominently upregulated on day 7 upon Bleomycin with a tendency to slightly decrease over time showed strong association with innate immunity (neutrophils, macrophages) and remodeling (collagen formation, ECM organization), with the top 3 genes being Sprr1a, Ccl2 and Ccl7. Pulmonary Sprr1a has previously been shown to be induced by inflammation and smoke^13^, while Ccl2/Mcp-1 and Ccl7 are well-known monocyte attractors. Interestingly, many of the genes in this cluster were also found upregulated upon TGFβ1 overexpression, in particular on day 21. A cluster of genes significantly altered upon AAV-TGFβ1, especially on day 14, was strongly associated with cell cycle processes, likely indicative of the immune cell activation and influx that was also evident from increased BAL cell counts at this time point (Fig. 1c–f). Saa3, Slc26a4 and IL-6 were the top 3 genes in this cluster. Saa3 has been shown to be required for lung development and homeostasis during lung inflammation and fibrosis^14^. Slc26a4/pendrin regulates HCO3^-^ efflux following cytokine stimulation, which enhances Cl^-^ secretion via CFTR, thereby impacting fluid and mucus homeostasis and was found upregulated in asthma and COPD^15,16^. Finally, IL-6 is a key pro-inflammatory cytokine orchestrating different immunological aspects, including neutrophil homeostasis, monocyte differentiation and T-cell activation. Genes that showed particularly strong induction on day 21 in the AAV model showed strong association with ECM organization and collagen formation, with Chl1, Timp1 and Lce3f being the top upregulated genes. The cell adhesion molecule Chl1 had not been described in the context of fibrosis so far but is thought to be a tumor suppressor and potential biomarker for survival in lung cancer^17^. Timp1 is a strongly fibrosis-associated inhibitor of matrix metalloproteinases (MMPs)^18^; in contrast, no lung or fibrosis association was found in literature for Lce3f. Similar pathways as the ones on day 21 were also found enriched for genes most strongly upregulated on day 28. The top genes in this cluster were Thbs4, Ph4a3 and Spp1. Thrombospondin 4 has been described as a pro-inflammatory and remodeling-associated protein in cardiovascular disease, among others^19^. Prolyl-hydroxylase 4a3 has more recently been described as a TGFβ1 downstream target with elevated expression in murine and human IPF^20^. Finally, Spp1/osteopontin is a prominent protein that is upregulated in various inflammatory and fibrotic conditions in the lung and other organs. Most of cluster 4 and 5 genes were also found induced in the Bleomycin model during day 7 and 28.

### Analysis of common and model-specific pathways and regulators

To further increase our understanding of the pathways altered by Bleomycin or TGFβ1, we took three different approaches. First, we assessed KEGG pathway enrichment for genes deregulated either specifically in one of the models or commonly in both models, defined by expression cutoffs (Fig. 2e). This approach confirmed the above findings by revealing day 14 as the major time point of DNA replication/cell proliferation in the AAV model as compared to biphasic cell cycle activity in the Bleomycin model (Fig. 2f). Early inflammatory processes were found to be unique to the Bleomycin model, while inflammation is also present in the AAV model, however, occurring rather simultaneously with fibrosis development between day 14 and 28.

Second, in order to identify genes altered specifically during a given disease stage, we pre-defined expression patterns and ranked genes by the degree of correlation between their longitudinal gene expression profiles and these patterns. Well-correlating hits were then applied to pathway analysis (Suppl. Fig. 4). Besides confirming the strongly Interferon/TLR-associated DNA damage events in the Bleomycin model on day 3, the analysis revealed many immunoglobulin genes among the most highly expressed genes specifically upregulated on day 21 and 28 in both models (Suppl. Fig. 4). Main phases of cell cycle activity (proliferation) were confirmed to occur on day 7 in the Bleomycin model and on day 14 in the AAV model. The function of downregulated genes became less clear; yet, in the AAV model, the data suggested that epithelial integrity (cilia and cell development) was impaired, starting from day 7. The continuously decreasing expression of surfactant proteins SP-A, SP-B and SP-C over time is in line with this finding (Suppl. Fig. 5). Interestingly, three genes associated with the GO term “negative regulation of osteoblast differentiation”, were specifically upregulated in the Bleomycin model on day 28, namely Chrd, Rorb and Limd1. Chordin is a developmental protein that binds bone morphogenic proteins (BMPs)^21^, while Lims domain containing 1 is regulated by tension and cell density to modulate Hippo/YAP and Wnt signaling^22^, possibly suggesting anti-fibrotic activity at this late time point.

**Figure 3:**
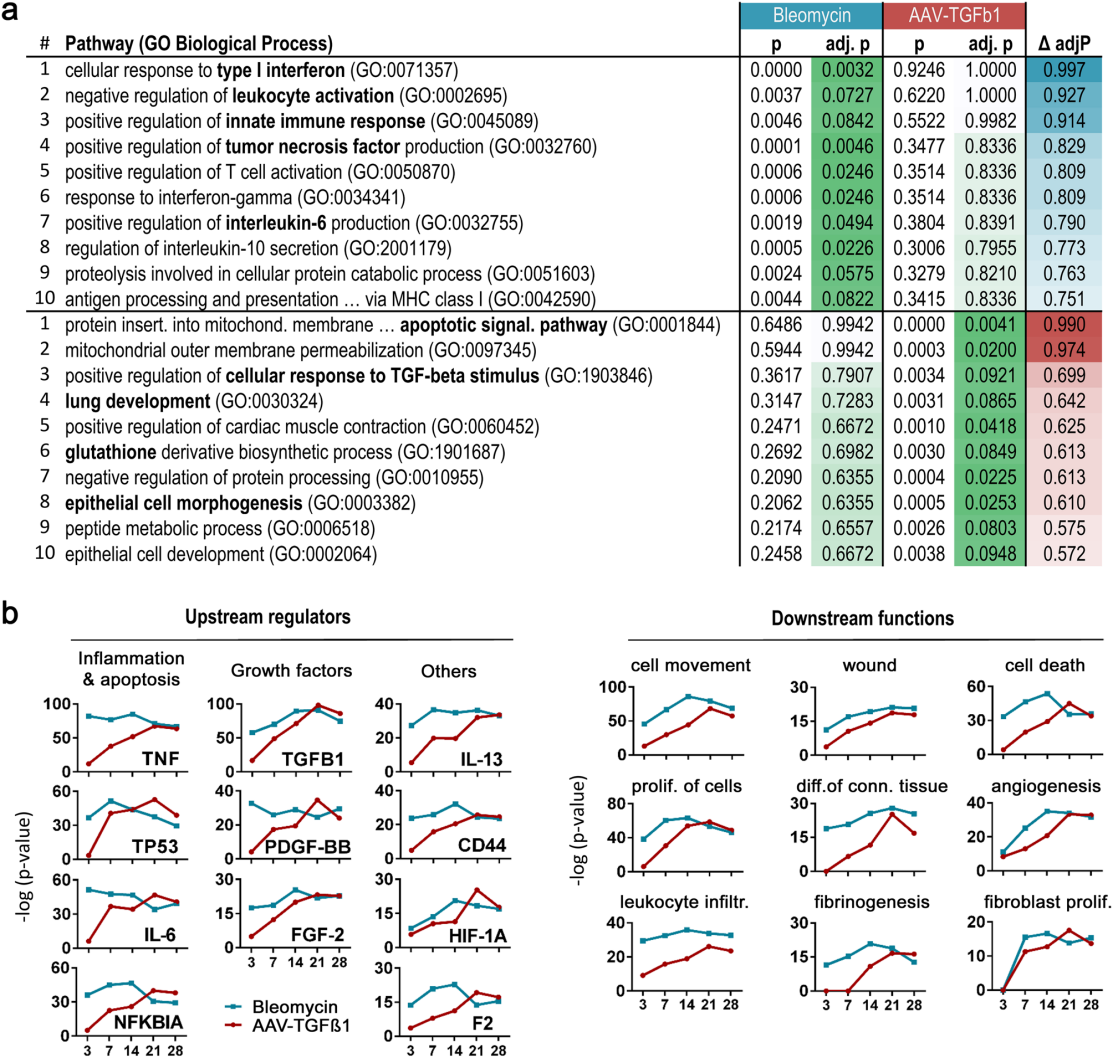
Differentially activated pathways and upstream regulator prediction. (**a**) Shown are the top 10 pathways preferentially enriched in the respective model, defined and ranked by the difference in pathway enrichment score (Δ adj. *p*) between the models. Color shades from dark to light green visually highlight *p*-values. (**b**) Selected, categorized upstream regulators (left) and downstream functions (right) as predicted by Ingenuity Pathway Analysis for all differentially expressed genes per time point.

Third, to further understand changes specifically occurring in one of the models, we selected all genes that were differentially expressed at least at one time point per model and applied them to pathway enrichment analysis. Following export of all pathways containing one or more of these genes, we ranked them by the difference in significance of enrichment (delta adj-p), i.e., we selected those pathways that showed strong enrichment in one but not the other model. The top 10 pathways preferentially altered in each model are depicted in Fig. 3a. For Bleomycin, pathways related to interferon-signaling, inflammation (TNF, IL6) and proteolysis/antigen processing were identified, mainly due to alterations in interferon-associated, TLR, cathepsin and proteasome genes (Suppl. Fig. 6). Preferentially altered pathways in the AAV model included apoptosis, developmental, glutathione- and epithelium-related pathways, all of which can be linked to known TGFβ functions. Not surprisingly, the term “positive regulation of cellular response to TGF-beta stimulus” showed particularly strong enrichment in the AAV model. Prominent genes in those pathways, among others, include BAX, BAD, VEGF, FGF and LOXL members, cyclin-dependent kinases and glutathione-transferases (Suppl. Fig. 6).

In a final approach, we used Ingenuity Pathway Analysis to predict upstream regulators and downstream functions and plotted the enrichment of selected candidates over time (Fig. 3b). These analyses showed strongest enrichment for TNF in the Bleomycin model, underscoring its early inflammatory character, whereas TGFβ was correctly predicted for the TGFβ model. The predicted downstream functions are well in line with the results of our previous analyses and provide additional insight into the time course of critical events, such as wounding, cell death and fibroblast proliferation.

Taken together, our results illustrate distinct differences in kinetics and early events during disease onset in AAV-TGFβ1- and Bleomycin-induced pulmonary fibrosis. At the same time, they also highlight striking similarities during the phase of fibrosis and tissue remodeling at later disease stages, in particular on day 21. Notably, while Bleomycin-induced fibrosis represents a biphasic response (acute inflammation followed by fibrogenesis), it appears that inflammation is evident to a similar degree in the TGFβ1 model, however, occurring rather simultaneously with fibrogenesis.

### Identification of genes central to experimentally induced fibrosis

**Figure 4:**
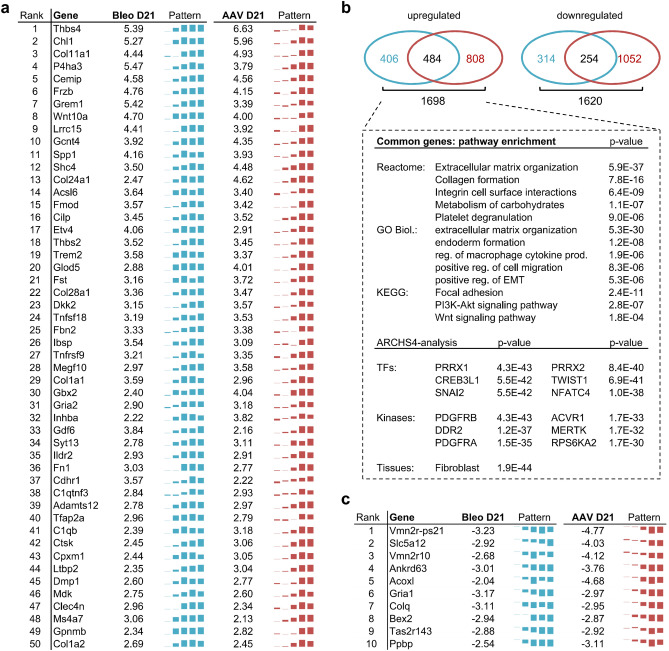
Genes associated with lung function decline. mRNAs of importance to fibrosis were identified by correlating their longitudinal gene expression changes to the decline in lung function observed in the respective model. (**a**) The top 50 highest expressed mRNAs showing strong anti-correlation to lung compliance (Pearson r ≤ − 0.85) and significant alteration (adj. *p*-value ≤ 0.05 at day 21 in both models) with consistent direction of expression (mean log_2_FC ≥ 0 in both models) are shown. log_2_ fold changes on day 21 are depicted together with the gene expression profile over all five time points. A list of all lung function (anti-) correlated transcripts, including correlation coefficients is supplied as Suppl. Table 2. (**b**) Venn diagrams of mRNAs showing strong lung function (anti-)correlation (threshold defined as |Pearson r|≥ 0.85) in either only one (blue = Bleomycin, red = AAV-TGFβ1) or both models. EnrichR analysis of Reactome, KEGG and GO biological pathways as well as ARCHS4 transcription factor co-expression, kinase co-expression and tissue predictions for all commonly lung-function anti-correlated genes. (**c**) Top 10 lung function-correlated (i.e. downregulated) mRNAs identified using the criteria from (**a**), adapted for positive correlation.

The remarkably similar phenotype in AAV-TGFβ1- and Bleomycin-induced fibrosis—despite two vastly different modes of disease induction (injury vs. persistent exogenous TGFβ1 expression)—suggests that certain underlying transcriptional alterations are required for fibrosis development, independent of the mode of disease induction. To identify genes of particular importance during pathogenesis, we focused on genes whose longitudinal expression changes strongly correlated with the observed decline in lung function (Fig. 1i), thereby linking transcriptomic to phenotypic changes. Genes whose expression changes closely paralleled the decrease in lung function (absolute correlation coefficient of ≥ 0.85) were selected and ranked by their mean fold change in expression on day 21 across both models (Suppl. Table 2). The top 50 upregulated (i.e., lung function anti-correlated) and top 10 downregulated (i.e., lung function correlated) genes are presented in Fig. 4a and c, respectively. Known protein interactions from STRING are also illustrated for the top 200 upregulated genes (Suppl. Fig. 7). Notably, besides a multitude of well-known fibrosis-associated hits, also several genes described more recently in the context of fibrosis were contained in the list, e.g., P4HA3 (rank 4), IBSP (rank 26) and CTHRC1 (rank 58). P4HA3, as already described above, is a TGFβ1-inducible subunit of collagen prolyl hydroxylase whose inhibition had some anti-fibrotic effects^20^ and IBSP was reported as part of a new 9-gene BAL biomarker set predictive of mortality in human IPF^23^. Finally, CTHRC1 might possibly be of use to stratify IPF patients^24^ and has recently been described as a marker of a cell population producing particularly high levels of collagen in murine and human IPF^25^.

Not surprisingly, when applying the 484 commonly upregulated, lung function decrease-associated genes to EnrichR pathway analysis, highly significant enrichment of ECM, focal adhesion, EMT and Wnt signaling associated processes was found (Fig. 4b). Consistently, fibroblasts were predicted as the top “ARCHS4 tissues” hit. Transcription factor co-expression analysis revealed PRRX1, CREB3L1 and SNAI2 (= Slug) as the top hits. Interestingly, a role for PRRX1 in driving fibroblast and hepatic stellate cell activation currently emerges for fibrotic diseases in the lung, skin and liver, respectively, and might therefore be an interesting therapeutic target^26–28^. Moreover, CREB3L1 was proposed to be critical for TGFβ1-mediated collagen induction^29^ and SNAI1/2 transcription factors are well-described in the context of EMT^30^, although their exact roles and interplay are yet to be worked out^31^. Most strikingly, ARCHS4 kinase co-expression analysis identified PDGF receptors A and B as well as DDR2 as the top hits. While PDGF receptors are one of the main targets of Nintedanib^32,33^, a role for DDR2 as a promising target in IPF has only emerged lately^34,35^. Finally, the upregulation of the top 15 genes (Fig. 4a) was also validated in a follow-up in vivo study by qPCR, clearly underscoring the robustness of our findings (Suppl. Fig. 8). Collectively, these results strongly suggest that our gene selection strategy yielded highly relevant candidates for biomarker and drug target identification.

### miRNA profiling and selection of candidates for exploratory drug research

**Figure 5:**
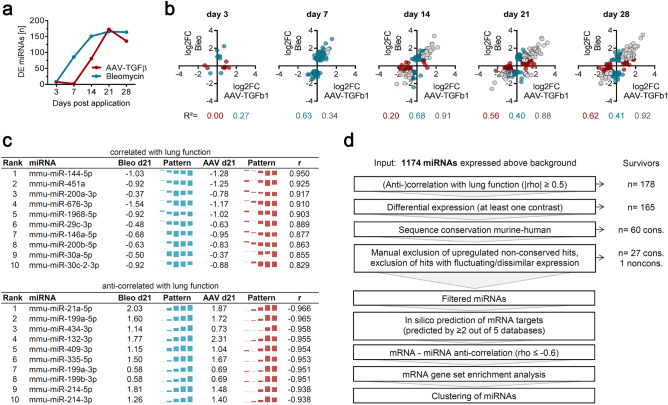
Longitudinal assessment of miRNAs and lung function association in AAV-TGFβ1 and Bleomycin induced fibrosis. Small RNAs were applied to next-generation sequencing analysis. (**a**) Number of differentially expressed miRNAs over time. (**b**) Correlation plots for miRNAs showing differential expression either exclusively in one model or commonly in both models, as defined by expression cutoffs (log_2_FC = 0.6). The coefficient of determination (R^2^) is show for each set of genes under the graphs. (**c**) The top 10 miRNAs most strongly anti-/correlated with the decline in lung function (Fig. 1i) are shown together with their log_2_ fold change in expression on day 21 and the expression pattern over all five time points. The mean Pearson correlation coefficient r over both models is also depicted. (**d**) Staged selection strategy to identify novel miRNA candidates for further characterization. rho = Spearman's rank correlation coefficient. For further details, see results and methods.

In addition to mRNA expression, we also analyzed changes in microRNA expression over time. Similar to the changes observed for mRNA, alterations in miRNA expression in the Bleomycin model occurred more rapidly with 86 differentially expressed (adj. *p*-value ≤ 0.05, |log2fc|≥ 0.6) miRNAs on day 7 as opposed to only two (non-overlapping) miRNAs in the AAV-TGFβ model at this time point (Fig. 5a,b). Also, correlation between the models was particularly strong during fibrosis development (d14-d28, Fig. 5b). Interestingly, despite the distinct TLR and interferon gene expression signature observed for Bleomycin on day 3, only few miRNAs were differentially expressed at this earliest time point. In contrast, highest deregulation and a significant overlap between the models was again observed on day 21 (Fig. 5a,b).

As for mRNA, we identified relevant miRNAs by correlating their longitudinal expression with the observed decrease in lung function over time. Assuming canonical miRNA function, lung function correlation (i.e., miRNA downregulation in parallel to the decrease in lung function) was expected to preferentially yield miRNAs, whose targets exert pro-fibrotic functions. In turn, miRNAs that are upregulated (i.e., anti-correlated to the decrease in lung function) should have targets with anti-fibrotic properties, whose suppression might therefore enable disease development. The top 10 lung function-correlated and -anti-correlated miRNAs are depicted in Fig. 5c. The deregulation of these miRNAs was also confirmed in a follow-up in vivo study by Nanostring nCounter measurements (Suppl. Fig. 9), thereby proving both, technical and biological validity of our findings.

Strikingly, the two top anti-correlated (i.e., upregulated) miRNAs identified using this approach were miR-199a-5p and miR-21a-5p (r = − 0.97), which are among the most well-known microRNAs associated with fibrotic disorders (Fig. 5c). miR-199 is overexpressed in several fibrotic diseases, found elevated in human IPF serum and exerts pro-fibrotic actions by suppressing calveolin-1, which increases TGFβ signaling^36,37^. miR-21a-5p is induced by TGFβ1 and further promotes its signaling by targeting the inhibitory SMAD7 in a feed-forward regulatory loop, thereby promoting fibroblast proliferation and fibrosis^38^. Many additional fibrosis-associated miRNAs were also identified; for instance, reasonably correlated with lung function (r = 0.69), we identified miR-29a-3p, which is a well-studied suppressor of many fibrotic genes^39^ and a potential therapeutic, as demonstrated by miR-29 mimics that were successfully used to attenuate fibrosis in vivo^40^.

**Figure 6:**
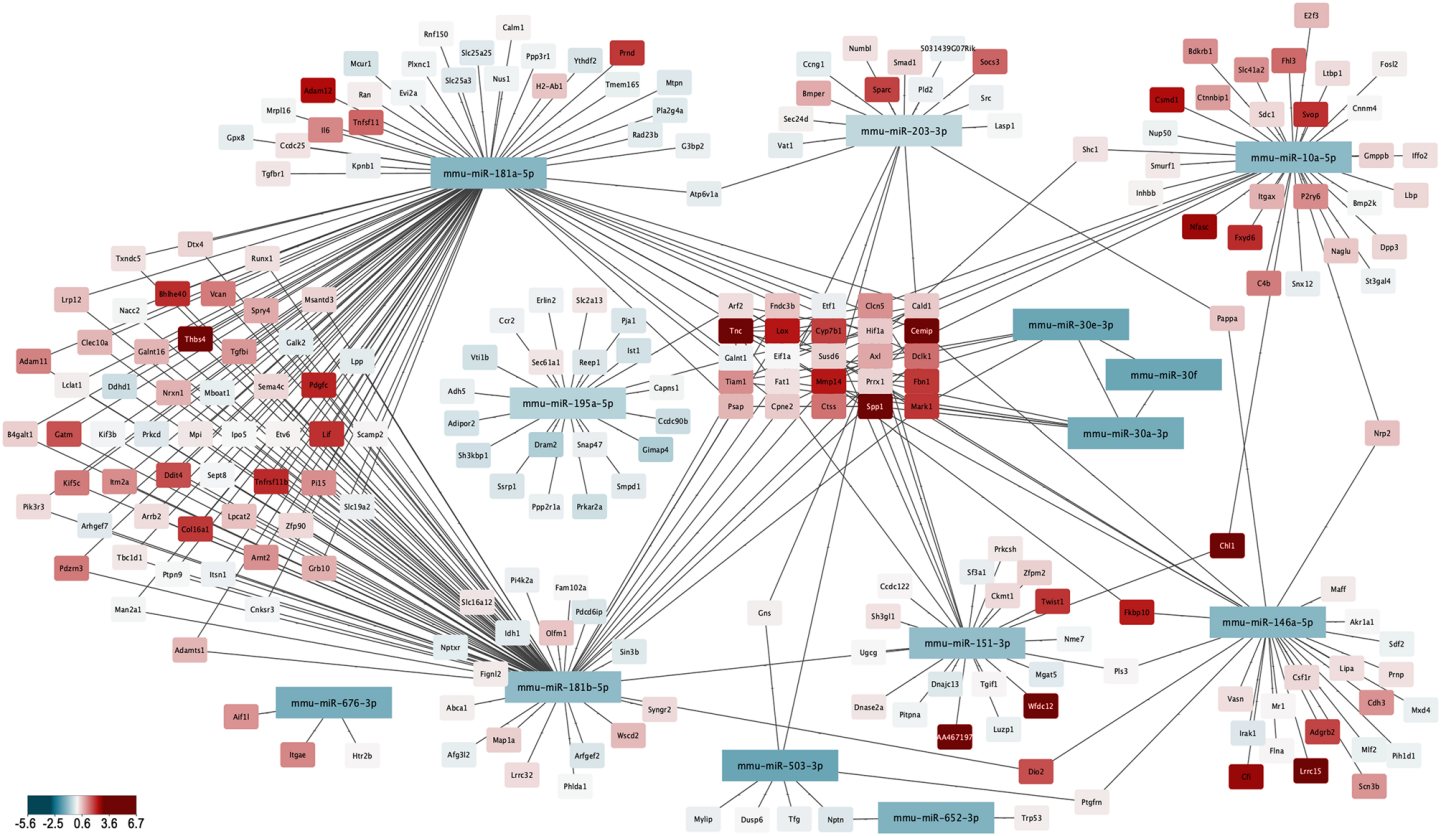
Network of all conserved downregulated miRNAs and their predicted DE targets. MicroRNAs were selected as described in Fig. 5d, with additional nodes selected as first neighbors of the selected miRNAs. Color scale of the node is the log2-fold change at day 21 in the AAV6.2-TGFβ1 model, with red indicating upregulation, and blue indicating downregulation. MiRNAs are represented as large rectangles, and mRNAs as small rectangles. Image created with Cytoscape 3.8.1.

We finally aimed to characterize less well-known microRNAs and their regulatory environment in more detail. To this end, we developed a network, integrating mRNA and miRNA expression data, miRNA target predictions queried through the multiMiR package^41^ and correlation data for all combinations of phenotypic lung function decline, longitudinal mRNA and longitudinal miRNA expression. A staged selection strategy (Fig. 5d) was then applied to identify miRNA candidates with the potential for therapeutic applications. Briefly, following selection for (anti-)correlation with lung function and differential expression, candidates were evaluated for murine-human species conservation, defined as perfect seed region conservation and at least 20 matched base pairs in the mature miRNA sequence. Upregulated, non-conserved candidates were excluded (as they cannot be targeted in humans) along with candidates that showed inconsistent deregulation across the models. Finally, to functionally classify potential mRNA targets (predicted in silico by at least 2 out of 5 prediction tools), they were analyzed for gene set enrichment (Suppl. Fig. 10). The resulting list of 16 up- and 12 down-regulated miRNAs is shown in Table 1. Moreover, a network of all downregulated candidates, including predicted mRNA targets that showed expression anti-correlation, is provided in Fig. 6.

**Table 1:** Hit list of lung-function associated miRNAs.

miRNA	DE	Sequence (mature miRNA)	miRNA	DE	Sequence (mature miRNA)
mmu-miR-501-3p	UP	AAUGCACCCGGGCAAGGAUUUG	mmu-miR-181a-5p	DOWN	AACAUUCAACGCUGUCGGUGAGU
mmu-miR-340-3p	UP	UCCGUCUCAGUUACUUUAUAGC	mmu-miR-10a-5p	DOWN	UACCCUGUAGAUCCGAAUUUGUG
mmu-miR-378a-3p	UP	ACUGGACUUGGAGUCAGAAGG-	mmu-miR-181b-5p	DOWN	AACAUUCAUUGCUGUCGGUGGGU
mmu-miR-1247-5p	UP	ACCCGUCCCGUUCGUCCCCGGA	mmu-miR-652-3p	DOWN	AAUGGCGCCACUAGGGUUGUG
mmu-miR-342-3p	UP	UCUCACACAGAAAUCGCACCCGU	mmu-miR-146a-5p	DOWN	UGAGAACUGAAUUCCAUGGGUU
mmu-miR-148a-3p	UP	UCAGUGCACUACAGAACUUUGU	mmu-miR-151-3p	DOWN	CUAGACUGAGGCUCCUUGAGG
mmu-miR-369-5p	UP	AGAUCGACCGUGUUAUAUUCGC	mmu-miR-195a-5p	DOWN	UAGCAGCACAGAAAUAUUGGC
mmu-miR-410-3p	UP	AAUAUAACACAGAUGGCCUGU	mmu-miR-503-3p	DOWN	GAG-UAUUGUUUCCACUGCCUGG
mmu-miR-431-5p	UP	UGUCUUGCAGGCCGUCAUGCA	mmu-miR-203-3p	DOWN	GUGAAAUGUUUAGGACCACUAG
mmu-miR-148b-3p	UP	UCAGUGCAUCACAGAACUUUGU	mmu-miR-676-3p	DOWN	CCGUCCUGAGGUUGUUGAGCU
mmu-miR-212-3p	UP	UAACAGUCUCCAGUCACGGCCA	mmu-miR-7656-3p	DOWN	ACAGGCUGUCUGAUCCCACGGU
mmu-miR-183-5p	UP	UAUGGCACUGGUAGAAUUCACU	mmu-miR-30f	DOWN	GUAAACAUCCGACUGAAAGCUC
mmu-miR-411-5p	UP	UAGUAGACCGUAUAGCGUACG			
mmu-miR-127-3p	UP	UCGGAUCCGUCUGAGCUUGGCU			
mmu-miR-212-5p	UP	ACCUUGGCUCUAGACUGCUUACU			
mmu-miR-146b-5p	UP	UGAGAACUGAAUUCCAUAGGCU

MicroRNAs whose expression continuously decreases with increasing disease severity, are likely to either be pro-fibrotic molecules that are suppressed in attempt to fight fibrosis or—maybe even more likely—suppressors of pro-fibrotic targets whose expression increases with worsening disease. Indeed, recent literature for the top downregulated hit, miRNA-181a-5p, shows that TGFβ1-induced proliferation and collagen production in a cellular model of hepatic stellate cells (LX2 cells) was abrogated by miR-181a-5p mimics^42^. A further report suggests that its inhibition upregulates IGF1 and WISP1 in an ex vivo cystic fibrosis model^43^, both of which are involved in wound healing and fibrosis. Therefore, these data suggest that (over)expression of miR-181a-5p might be a strategy to suppress fibrosis. This example, together with the successful identification of highly fibrosis relevant genes by the application of a similar phenotype-transcriptome selection approach for mRNAs (Fig. 4) suggests that the hit list (Table 1) is a very attractive starting point for the identification of therapeutically relevant miRNAs.

## Discussion

The Bleomycin-induced lung injury model is undoubtedly the most broadly used model to study lung fibrosis in mice. However, as every mouse model of human disease, also the Bleomycin model is limited in its ability to mimic all relevant aspects of human lung fibrosis. In this regard, two debatable aspects are its mode of action (MoA) based on acute lung injury and its usually transient nature. As an alternative model lacking these features and instead focusing largely on the actual fibrotic aspect of the disease, we developed the AAV-TGFβ1 model^5^. Because AAV-mediated gene transfer does not elicit strong side effects like, e.g., anti-viral immune responses or direct cytotoxicity, the phenotype observed in this model is solely induced by the overexpressed transgene—TGFβ1—which, due to AAV6.2’s cellular tropism, is persistently expressed from bronchial epithelial and alveolar type II cells. Therefore, continuous TGFβ1 expression is achieved, which leads to a progressive fibrotic phenotype (Fig. 1).

While our first AAV-TGFβ1 study established proof-of-concept for fibrosis induction and its dose-dependency, this current work aimed to delineate the details of TGFβ1- and Bleomcyin-induced fibrosis in order to (1) understand the differences and commonalities on a phenotype, gene and pathway level to enable proper model selection depending on the scientific question of interest; (2) reveal relevant time points for pharmacological intervention studies, dependent on the MoA of respective compounds; (3) identify drug target and biomarker candidates as starting points for future therapeutic concept research; (4) built hypotheses on disease-relevant miRNA-mRNA interactions and (5) generate a publicly available data resource for future target/pathway-specific questions.

The main difference between the two models clearly lies in the acute injury component of the Bleomycin model, evident from the observed innate immune response, increase in BAL protein (due to epithelial damage) and resulting loss in body weight. Corresponding transcriptomics also indicated activation of the clotting cascade, TLR signaling and a distinct type I interferon signature, in line with previous observations^44^. A link between DNA damage and subsequent interferon signaling has been established^45,46^, which likely explains the activation of this process upon instillation of DNA-damaging Bleomycin^47^. In contrast, the AAV-TGFβ1 vectors do not induce tissue damage upon instillation and no inflammation was therefore observed during the first week after treatment, which was supported by the very low number of differentially expressed genes at day 3 and 7 and no association to inflammatory or anti-viral processes. Nevertheless, upon TGFβ overexpression, monocyte/macrophage and neutrophil BAL counts were similarly high as after Bleomycin treatment, in line with literature showing that TGFβ, in the absence of cytokines or TLR ligands, stimulates myeloid cell proliferation and inflammatory cytokine secretion^48^. Notably, while treatment with an ALK5 inhibitor from day 7 fully blocked fibrosis development, immune cell counts were reduced to a lesser degree (Suppl. Fig. 2), suggesting that early TGFβ effects are sufficient to induce inflammation, whereas subsequent fibrogenesis requires continuous TGFβ signaling. Overall, our study clearly shows a biphasic response in the Bleomycin model, with acute injury and inflammation leading to subsequent fibrosis, contrary to simultaneously occurring inflammation and fibrogenesis in the AAV-TGFβ model.

Given that we found an overall higher number of AAV-TGFβ1 exclusive genes, we wondered what processes would be specifically altered in this model. The different approaches taken to answer this question mainly revealed apoptosis, prominent cell cycle activation, alterations in glutathione metabolism and a more pronounced decrease in surfactant protein expression. Glutathione is known to be decreased by TGFβ1 and decreased levels are also found in fibrotic diseases^49^. Apoptosis, cell cycle activation and a decrease in surfactant proteins are perfectly in line with the presumed molecular pathogenesis of IPF, i.e., a perpetuation of epithelial cell apoptosis and proliferation as a consequence of an initial insult and subsequent pro-fibrotic stimuli^50^. Finally, two major differences between the models are first, the histological presentation of fibrosis, which appears patchy and bronchocentric in the Bleomycin model, due to its MoA based on direct injury that is in contrast to AAV6.2-mediated, bronchial epithelium- and AT2-cell specific expression of TGFβ, which leads to more evenly distributed fibrosis, reminiscent of the histopathological features of fibrosing non-specific interstitial pneumonia (NSIP)^10^. Second, less fluctuation and continuously worsening fibrosis with the AAV approach as compared to Bleomycin-treated animals, which show higher variability due to the randomly distributed injury events and resulting differences in the timing and extent of regeneration.

Despite the obvious and expected differences between the models, a remarkably similar phenotype and overlap of deregulated genes was observed during the main fibrotic stage, in particular at day 21. Overlapping genes showed strong association with typical fibrosis-relevant pathways, such as ECM organization, inflammatory response, response to wounding, cell proliferation, etc. However, in addition to this static analysis, we aimed to take into account both, the longitudinal effects and their functional/physiological consequences. Therefore, relevant mRNAs and miRNAs were identified based on the strength of correlation between their longitudinal expression changes and the continuous decrease in lung function observed in both models. The validity of this approach was clearly proven by the presence of numerous well-known fibrosis-associated genes (e.g., thrombospondins, osteopontin, collagens, fibronectin, gremlin and Wnt members) and miRNAs (e.g., miR-21, miR-199a) among the top hits. More importantly, besides those well-known genes, also several only recently emerged fibrosis-associated genes, such as P4HA3, CTHRC1 and FMOD^20,24,25,51^ were rapidly identified by our approach. Similarly, pathway, transcription factor and kinase upstream predictions using ARCHS4 suggested well-known (e.g., PDGFR, Slug, Twist) and novel regulators as pathogenic drivers, such as DDR2^34,35^. Given that our miRNA selection approach included several additional layers, i.e., mouse-human conservation, mRNA target prediction and anti-correlation thereof, we consider the resulting hit list, which comprises 16 upregulated and 12 downregulated miRNAs a very attractive basis for the investigation of their anti-fibrotic potential in human disease. From a drug development point of view, microRNAs represent a particularly attractive molecule class, due to their ability to control and fine-tune entire signaling pathways or cellular mechanisms. In fact, the therapeutic effect of Nintedanib, a triple receptor kinase inhibitor of VEGF, PDGF and FGF^33^ in comparison to largely inefficacious single targeting approaches pursued in the past, suggests that polypharmacological treatment strategies might be necessary to achieve therapeutic benefit in the complex pathology of pulmonary fibrosis^52^.

Our study adds a valuable data set to the existing collection of transcriptome data of murine lung fibrosis models, which so far mostly focused on the Bleomycin model^53–55^ or cellular isolates thereof^56^. Advantages of our study include the use of RNA-sequencing (as opposed to microarrays^53,54^), a minimally immunogenic vector system enabling the study of direct and persistent TGFβ effects, combined phenotypic, mRNA and miRNA measurements, and longitudinal sampling. The main limitation is the use of bulk lung RNA (extracted from flushed lung tissue), which usually achieves higher sequencing-depth than single cell-seq but is limited in cellular resolution. While this is sufficient for the characterization of many pathological processes, some specific aspects can only be addressed using technologies that provide information on the cellular level, including single-cell sequencing, but also histology or FACS. One example in that regard is aging, which is likely to play a major and possibly causal role in the development of pulmonary fibrosis^57^. One hallmark of aging is cellular senescence, which is characterized by DNA double-strand breaks, telomere shortening, cell cycle arrest and a senescence-associated secretory phenotype (SASP), characterized by proteins including PAI1, MMPs, IL6, IL8, MCP1, PDGF and TGFβ^57,58^. While DNA and telomere alterations cannot be assessed by bulk RNA-sequencing, many of the mentioned SASP- and other cellular senescence-associated genes (e.g., histone and cyclin dependent kinase genes) were found deregulated in our data. Yet, it cannot be easily concluded that this is indeed due to senescence, as those proteins can be secreted by several different cell types. Therefore, aging is one important disease-contributing factor that requires studies with cellular resolution. Notably, there is a rapidly growing number of single-cell studies in the field of lung fibrosis^59–64^, and first examples for the integration of transcriptomic and proteomic data^55,65^ as well as a rising number of metabolomics and lipidomics studies, including one that compared young and aged mice^66^, all of which steadily increase our understanding of the complex interplay and levels of regulation during PF pathogenesis. Still, given that all these studies made use of the Bleomycin model, we consider our AAV-TGFβ model and data a very valuable addition to the field.

As with any disease related animal model, a key question is to what extent the AAV-TGFβ1 model recapitulates human IPF/PF-ILD features, especially when compared to the widely used Bleomycin model. While we refrain from a general answer to this question, due to our conviction that it can only be answered on a pathway level, a strategy to assess this aspect in the required level of detail has been developed in our group. This computational approach, termed “In Silico Treatment” assesses translation of disease-related molecular expression patterns between animal models and humans and simulates experimentally observed expression changes on human data sets to quantify their impact on disease outcomes. The respective manuscript, covering a deepened analysis of this current data set in terms of human disease-relevance, is currently in preparation (Picart-Armada et al.). In general, to improve backtranslation into disease models, high quality and well-annotated clinical IPF datasets are still desperately needed, given that the number of available studies and respective patient samples is low (reviewed in^67^).

Furthermore, in addition to models like Bleomycin-induced lung injury that aim to broadly recapitulate human disease phenotypes in mice, we see particular value in using AAVs for the targeted modulation of pathways of interest, enabling a more mechanistic understanding of the mode of action of certain proteins of interest. In this context, AAV’s low immunogenicity in mice is an important property, as it allows for specific modulation of pathways without interference by vector-targeted immune responses. Since its development, the AAV-TGFβ1 model has been frequently used to study drug candidates and explore target function in our group^68–70^. Further technological refinement of the model might be achieved in the future by using self-complementary AAV vectors, which lead to a faster onset of transgene expression^71^. Moreover, our previously obtained dose–response data suggest that further fine-tuning of the AAV dose enables establishing fibrosis at a milder degree that might allow studying disease progression beyond four weeks, which was limited in the current study due to the severe effects on body weight at late time points (Fig. 1). Finally, the implementation of gene regulatory elements, such as artificial riboswitches, was shown to increase AAV-TGFβ1 vector production^11^ and could potentially be used to regulate AAV-mediated TGFβ1 expression using small molecules^72^.

In summary, our study provides a detailed characterization of the pathogenic processes underlying AAV-TGFβ1-mediated lung fibrosis development in comparison to the well-established Bleomycin lung injury model. The integration of longitudinal transcriptome and lung function data enabled the identification of mRNA-miRNA networks directly associated with disease development in mice. Our data will therefore be of value for drug discovery and biomarker research in the context of progressive fibrosing interstitial lung disease.

## Methods

### AAV vectors

Expression constructs harboring the murine TGF-β1 cDNA (NCBI Ref Seq NM_011577.2) with the constitutively activating mutations C223S and C225S^73^ under the control of a CMV promoter or non-coding “stuffer” DNA^5^ were synthesized at Life technologies and cloned into a pAAV vector harboring AAV2 ITR sequences. For the production of recombinant AAV6.2 vectors, the AAV2 cap gene in pAAV-RC (Agilent Technologies, Waldbronn) was replaced by the AAV6.2 cap gene (GenBank: EU368910.1). HEK-293 cells were triple-transfected with pAAV-AAV6.2cap, pHelper (Agilent Technologies) and either the TGFβ1 or stuffer constructs, using the calcium-phosphate transfection method. The vectors were purified using the iodixanol-based protocol described in detail before^74^.

### Animal studies

All animal experiments were approved by the local authorities (Regierungspräsidium Tübingen, Germany) and performed and reported in accordance with the German law on animal welfare and under consideration of the ARRIVE guidelines. Male 9–12 wk. old C57Bl/6 mice (120 in total) were obtained from Charles River Laboratories (Sulzfeld, Germany) and allowed to acclimate for one week prior to intratracheal administration of either 2.5 × 10^11^ vg of AAV-TGFβ1 or AAV-stuffer, 1 mg/kg Bleomycin or NaCl solution in a volume of 50 µL, which was carried out under light anesthesia (3–4% isoflurane). For pharmacological inhibition of the type I TGFβ1 receptor (ALK5), the inhibitor SB-525334 was diluted in 0.5% Natrosol and orally administered to the mice (28 in total) from day seven after AAV administration at 30 mg/kg in a once or twice daily dosing regimen. Body weight was monitored daily and animals with a loss of body weight greater than 20% were taken out of the experiment and euthanized. Fibrosis was assessed at day 3, 7, 14, 21 and 28 after AAV/Bleomycin administration and on day 21 in the ALK5 inhibitor experiment, respectively. Lung function, µCT, immune cell, histological and bronchoalveolar lavage (BAL) analyses were described in detail in one of our previous publications^5^. Briefly, for µCT imaging, mice were anesthetized using 4% isoflurane and kept under 1.5% isoflurane anesthesia during CT scanning, which was carried out on a Quantum FX µCT (Perkin Elmer). To assess lung function, mice were anesthetized by i.p. administration of pentobarbital/xylazine hydrochloride, cannulated intratracheally and treated with pancuronium bromide by i.v. administration. Lung function measurement was then conducted using the flexiVent FX system (Scireq). Mice were euthanized by a pentobarbital overdose, the lung was dissected and weighed prior to flushing with 2 × 700 µL PBS to obtain BAL fluid. Differential BAL immune cell counts were determined using the Sysmex XT1800 iVet cell analyzer and cytospins. BAL TGFβ1 levels were measured using the Mouse TGF-beta 1 Quantikine ELISA (#MB100B, R&D Systems, Minneapolis, MN) according to the instructions provided with the kit. The right lung of each mouse was processed for histological assessment by a histopathologist according to the Ashcroft criteria^75^. The left lung was used for total RNA extraction, as detailed below.

### RNA preparation

For total lung RNA preparation, the left lung was flash frozen in liquid nitrogen immediately after dissection. Frozen lungs were homogenized in 2 mL precooled RLT buffer (Qiagen, Hilden, Germany) + 1% β-mercaptoethanol (Sigma-Aldrich) using the Peqlab Precellys 24 Dual Homogenizer and 7 mL-ceramic bead tubes (#91-PCS-CK28L, Peqlab, Erlangen). 150 µL homogenate were then mixed with 550 µL QIAzol Lysis Reagent (#79306, Qiagen). After addition of 140 µL chloroform (Sigma-Aldrich), the mixture was shaken vigorously for 15 s and centrifuged for 5 min at 12,000 xg and 4 °C. 350 µL of the upper aqueous phase (containing RNA) were then further purified using the miRNeasy 96 Kit (#217061, Qiagen) according to the manufacturer’s instructions. After purification, RNA concentration was determined using a Synergy HT multimode microplate reader and the Take3 module (BioTek Instruments, Winooski, VT, USA). RNA quality was assessed using the 2100 Bioanalyzer (Agilent Technologies).

### Library preparation and sequencing

cDNA libraries were prepared using the TruSeq Stranded Total RNA Sample Preparation Kit (Illumina, San Diego, CA, USA) and 200 ng of total RNA. Following purification and PCR enrichment of the cDNAs, the library was diluted to 2 nM and clustered on the flow cell at 9.6 pM, using the TruSeq SR Cluster Kit v3-cBot-HS (#GD-401-3001, Illumina) and the cBot instrument (Illumina). Sequencing of 52 bp single reads and seven bases index reads was performed on an Illumina HiSeq 2000 using the TruSeq SBS Kit v3-HS (#FC-401-3002, Illumina). Approximately 20 million reads were obtained per sample. For miRNA, the TruSeq Small RNA Library Preparation Kit was used (Illumina) to prepare the cDNA library: As a result of miRNA processing by Dicer, miRNAs contain a free 5’-phosphate and 3’-hydroxal group, which were used to ligate specific adapters prior to first and second strand cDNA synthesis. By PCR, the cDNAs were then amplified and indexed. Using magnetic Agencourt AMPure XP bead-purification (#A63881, Beckman Coulter), large DNAs were separated and small RNAs were enriched. Similar to mRNA, the samples were finally clustered at 9.6 pM and sequenced, while being spiked into mRNA sequencing samples.

### Read mapping and quality control

RNA-Seq reads were aligned to mouse genomes using the STAR Aligner v2.5.2a^76^ with their corresponding Ensembl 86 reference genomes^77^. Sequenced read quality was checked with FastQC v0.11.2 (http://www.bioinformatics.babraham.ac.uk/projects/fastqc/) and alignment quality metrics were calculated using the RNASeQC v1.1875. Following read alignment, duplication rates of the RNA-Seq samples were computed with bamUtil v1.0.11 to mark duplicate reads and the dupRadar v1.4 Bioconductor R package for assessment^78^. The gene expression profiles were quantified using Cufflinks software version 2.2.1^79^ to get the Reads Per Kilobase of transcript per Million mapped reads (RPKM) as well as read counts from the featureCounts software package^80^. Differential expression analysis was performed with the uniquely mapping read counts as input for the Bioconductor LIMMA analysis R package with voom normalization^81^. Descriptive analyses such as PCA and hierarchical clustering were carried out to identify possible outliers. Differential expression between treatment and respective controls at each time points were carried out using limma with a significance threshold of *p* adj ≤ 0.05 and abs(log2FC) ≥ 0.5. Two samples out of 120 in total were excluded for not passing QC criteria.

### miRNA-Seq reads

Sequenced read quality was checked with FastQC (FastQC version 0.11.2). Subsequently, miRNA-sequencing read adapters were detected using minion and trimmed using reaper from the kraken package, version 13–274^82^. The trimmed reads were aligned to the Ensembl v86 mouse genome using the STAR Aligner (version 2.5.2a) using the following parameters:—outFilterMismatchNoverLmax 0.05—outFilterMatchNmin 16—outFilterScoreMinOverLread 0—outFilterMatchNminOverLread 0—alignIntronMax 1 (parameters taken from personal communication with the STAR author A. Dobin). Aligned reads were filtered for mature miRNA lengths of between (or equal to) 16 and 26 bp in size. The SAM files from STAR were converted to BAM files using samtools 0.1.18^83^. The aligned miRNA reads were then quantified using subread from the featureCounts package (version 1.4.5-PR1)^80^ and the miRBase 21 as a reference^84^.

### Mouse-human conservation of miRNA sequences

For all murine and human miRNAs from miRBase 21 seed regions (position 2 to 7) were extracted. For all combinations of murine and human miRNAs global alignments between the seed regions and the mature were calculated using the pairwiseAlignment function from the Bioconductor Biostrings package (v2.46.0). We applied the Needleman-Wunsch algorithm using an RNA substitution matrix with a match score of 1 and a mismatch score of 0. We assigned two categories to the miRNA candidates—“conserved” for miRNAs with an alignment score of 6 in the seed region for mouse-human pairs of miRNAs with the same name, “non-conserved” for miRNAs with an alignment score < 6 in the seed region for mouse-human pairs of miRNAs with the same name. In addition, miRNAs with an alignment score for the alignment of the respective mature sequences above 20 is assigned to the category “mature high similarity”.

### Determination of putative miRNA-mRNA target pairs

To determine mRNA targets of miRNAs, a stepwise approach has been carried out. First, lowly expressed miRNAs and mRNAs were removed from the expression matrix. Subsequently, the Spearman’s rho was calculated between voom transformed log(CPM) of each miRNA vs. each mRNA across all samples of both models and all time points, using the corAndPvalue function from WGCNA v. 1.60^85^.The set of correlation based putative miRNA-mRNA pairs is defined as all combinations with a correlation ≤ − 0.6. To add sequence-based prediction of putative miRNA-mRNA pairs, all combinations with predictions in at least two out of five most cited miRNA target prediction algorithms (DIANA, Miranda, PicTar, TargetScan, and miRDB) available in the Bioconductor package miRNAtap v. 1.10.0 / miRNAtap.db v. 0.99.10 were taken as sequence-based pairs. The final set of miRNA-mRNA pairs is the intersection of anticorrelation based and sequence-based interaction pairs, reducing the number of predictions significantly to a high-confidence subset.

An integrated miRNA-mRNA network was built using the high-confidence miRNA-mRNA interactions as well has highly correlated mRNA-mRNA edges (correlation ≥ 0.75). Node annotation consists of number of contrasts with significant differential expression of the respective miRNA or mRNA and log-fold change in the various contrasts described above. In addition, each node is annotated with the correlation to the functional parameters. The network was imported into Cytoscape for node selection and visualization purposes.

### Pathway analysis

Pathway analyses for differentially expressed mRNAs were carried out using EnrichR^86,87^ and Ingenuity Pathway Analysis. For EnrichR analyses, gene lists obtained from either heatmap cluster extraction (Fig. 2b) or analyses of differences and commonalties between the models (Figs. 2f, 3a and 4b) were defined as described in the results section or Figure legends and subsequently applied as input search terms. Enriched Reactome^88^ and KEGG pathways^89^ as well as GO Biological Processes^90^, and ARCHS4^91^ transcription factors, kinases, and tissues, respectively, were then extracted from the respective EnrichR results sections. Selected pathways along with their corresponding adjusted p-values, which are calculated using the Benjamini–Hochberg method for correction for multiple hypothesis testing, are displayed in Figs. 2, 3 and 4. Ingenuity Pathway Analysis was used to predict putative upstream regulators and downstream functions. Upstream regulator prediction was limited to the categories cytokines, growth factors, kinases and others and restricted using p-value cutoffs of 3 (log10) and a Z-score cutoff of 2. From the resulting list of potential regulators, several were selected, and their enrichment p-values were plotted over time (Fig. 3b). The same approach was applied to identify downstream functions, using Ingenuity’s Diseases & Functions analysis.

### Statistics and data visualization

Log2-fold changes for Bleomycin samples were calculated relative to NaCl treatment, whereas AAV-TGFβ1 values were calculated relative to AAV-stuffer treatment. Genes with an absolute log2-fold change ≥ 0.6 and an adjusted *p*-value ≤ 0.05 were called differentially expressed, unless stated differently in the main text or figure legends. Statistical significance for phenotypic readouts (Fig. 1) was assessed by either one-way ANOVA and Sidak’s post-test or two-way ANOVA and Tukey’s post-test. *p < 0.05, **p < 0.01, ***p < 0.001. Data were^86,87^visualized using GraphPad Prism, Tibco Spotfire and Cytoscape.

## Supplementary Information

Supplementary Figures.

Supplementary Table 1.

Supplementary Table 2.

## Data Availability

Sequencing data have been deposited in NCBI’s Gene Expression Omnibus and are accessible through GEO Series accession number GSE195773 [https://www.ncbi.nlm.nih.gov/geo/query/acc.cgi?&acc=GSE195773].
