# Supplementary material for: Time and phenotype-dependent transcriptome analysis in AAV-TGFβ1 and Bleomycin-induced lung fibrosis models
Source: Sci Rep. 2022 Jul 16;12:12190. doi: 10.1038/s41598-022-16344-7 (PMC9288451; doi:10.1038/s41598-022-16344-7)
Supplement: Supplementary file 1 — Supplementary Figures. [file 41598_2022_16344_MOESM1_ESM.pdf]

Supplemental Information

Time and phenotype-dependent transcriptome analysis in AAV-TGFβ1 and Bleomycin-induced lung fibrosis models

Benjamin Strobel, Holger Klein, Germán Leparc, Birgit E. Stierstorfer, Florian Gantner, Sebastian Kreuz

Supplemental Figure 1

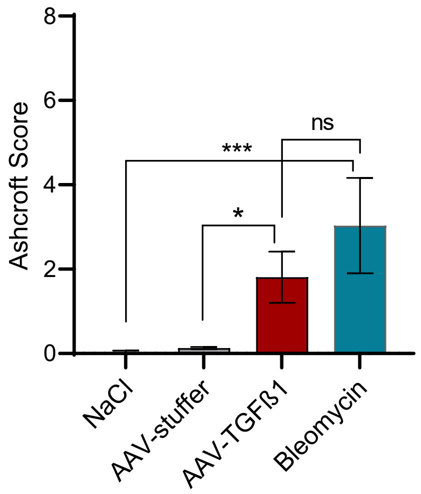

Suppl. Fig. 1: Ashcroft Score. Mice were treated as described in main Figure 1. The graph depicts the Ashcroft score obtained by assessment of histological sections at day 21 after AAV and Bleomycin administration, respectively, by a trained histopathologist. Mean±SD. *$p<0.05$, ***$p<0.001$. ns= not significant.

Supplemental Figure 2

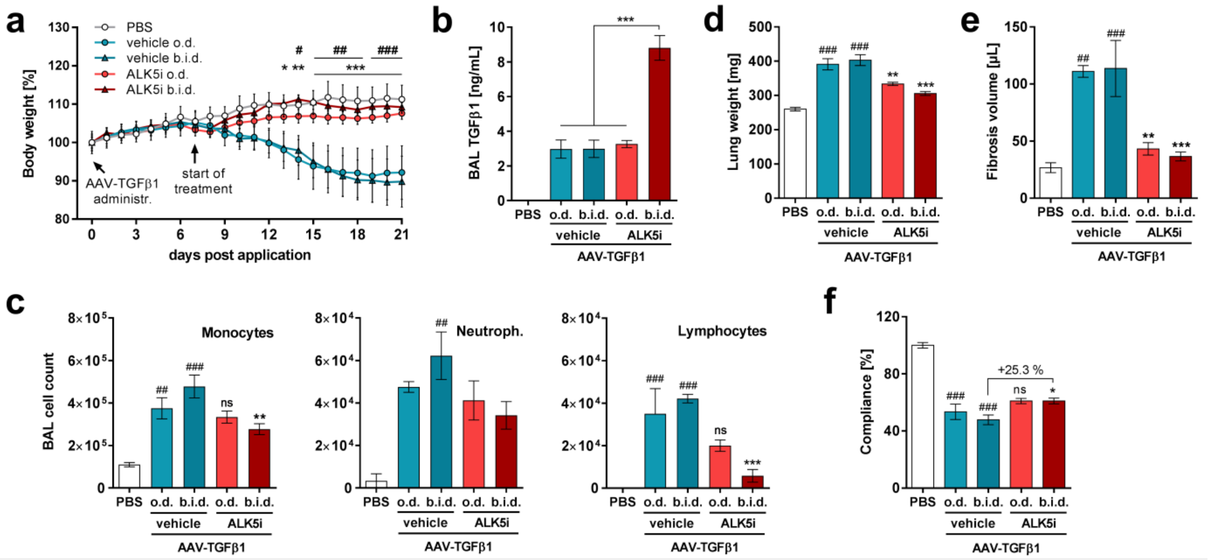

Suppl. Fig. 2: Treatment of AAV-TGFβ1 mice with the ALK5 inhibitor SB-525334. Mice either received PBS or 2.5x10[11] vg AAV-TGFβ1 by i.t. administration on day 0 and were treated with 30 mg/kg of SB-525334 or vehicle, starting day 7 by oral (gavage) application, either once (o.d.) or twice (b.i.d.) daily. (**a**) Body weight measurement. (**b**) TGFβ1 protein levels measured in BAL samples using ELISA. (**c**) Differential immune cell counts in BAL on day 21 post AAV application (i.e. ,14 days post compound treatment. (**d**) Wet lung weight. (**e**) micro-CT analysis of fibrotic lung volume. (**f**) Lung function (compliance) measurements. n= 3 (PBS), 4 (vehicle o.d.), 5 (vehicle b.i.d.) and 8 (ALK5i) animals per group. Mean +/- SEM. */# $p<0.05$, **/##$p<0.01$, ***/###$p<0.001$. Hashmarks and asterisks indicate statistical significance relative to PBS treatment and the respective vehicle control groups, respectively.

Supplemental Figure 3

a

	AAV-TGFβ1 vs. AAV-stuffer										Bleomycin vs. NaCl										
	log2FC					adj. P-value					log2FC					adj. P-value					FPKM
Gene	d3	d7	d14	d21	d28	d3	d7	d14	d21	d28	d3	d7	d14	d21	d28	d3	d7	d14	d21	d28	max
Col11a1	-0.29	-0.58	0.58	4.22	4.42	1E+00	1E+00	9E-01	1E-02	5E-03	0.97	0.34	5.01	5.79	6.40	7E-01	9E-01	2E-03	5E-04	9E-04	2.51
Col24a1	0.15	1.09	3.15	4.48	4.20	1E+00	6E-01	2E-04	1E-08	6E-08	1.25	2.47	3.84	3.50	3.84	9E-02	6E-05	2E-07	4E-06	3E-06	2.87
Col28a1	0.20	0.35	1.65	3.72	3.04	9E-01	9E-01	3E-03	2E-10	7E-11	1.20	2.24	3.27	3.16	3.46	3E-02	3E-07	5E-11	7E-09	5E-08	9.78
Col1a1	0.21	0.58	1.92	3.58	3.12	8E-01	4E-01	5E-08	3E-14	3E-16	1.11	1.97	2.98	2.97	2.50	4E-04	6E-11	2E-18	2E-16	3E-12	372.83
Col5a2	0.43	0.75	2.24	2.96	2.51	5E-01	2E-01	5E-11	2E-12	1E-13	1.21	1.93	2.58	2.57	2.48	1E-05	5E-12	1E-17	2E-15	2E-13	235.57
Col1a2	0.23	0.39	1.64	2.82	2.38	7E-01	5E-01	6E-09	8E-14	3E-15	0.92	1.63	2.26	2.34	2.15	2E-04	2E-11	1E-17	3E-16	1E-13	561.26
Col15a1	0.05	0.46	1.33	2.48	2.14	1E+00	6E-01	2E-04	1E-09	5E-10	0.84	1.78	2.38	2.42	2.08	2E-03	2E-09	2E-12	8E-11	9E-08	25.08
Col3a1	0.41	0.73	2.25	2.70	2.08	4E-01	7E-02	4E-15	2E-14	3E-14	1.29	1.76	2.22	2.17	1.81	2E-08	4E-14	4E-19	1E-16	2E-12	765.75
Col5a3	-0.41	-0.09	1.21	2.48	1.83	7E-01	1E+00	1E-02	2E-07	2E-05	0.94	1.63	2.53	2.29	2.13	1E-02	4E-06	2E-09	4E-08	4E-06	8.76
Col7a1	-0.54	0.04	0.91	2.50	1.90	6E-01	1E+00	5E-02	1E-07	2E-06	0.04	0.91	1.57	2.04	1.90	9E-01	2E-03	1E-06	7E-08	4E-06	8.25
Col5a1	0.05	0.39	1.26	2.34	1.88	1E+00	5E-01	2E-05	2E-10	2E-10	0.76	1.41	2.06	2.15	1.93	2E-03	6E-09	7E-15	9E-14	8E-11	51.09
Col8a1	-0.26	0.19	0.77	2.15	2.29	8E-01	9E-01	3E-02	1E-09	3E-13	0.86	0.68	1.87	2.23	2.49	5E-03	6E-03	3E-11	1E-12	6E-12	16.90
Col4a1	0.64	0.48	1.41	1.96	1.49	1E-01	3E-01	1E-08	3E-10	1E-09	0.85	0.99	1.46	1.56	1.41	5E-05	9E-07	5E-12	1E-11	2E-09	385.33
Col16a1	0.16	0.32	1.25	2.08	1.47	7E-01	4E-01	9E-09	3E-15	4E-12	0.48	0.69	1.03	1.36	1.36	4E-03	6E-06	4E-10	2E-12	7E-11	35.94
Col4a2	0.62	0.38	1.01	1.78	1.48	7E-02	3E-01	8E-07	2E-11	4E-12	0.52	0.69	1.18	1.32	1.19	4E-03	4E-05	4E-11	2E-11	3E-09	252.52
Col12a1	0.76	0.50	0.95	1.32	0.99	1E-01	4E-01	9E-04	7E-05	3E-04	0.59	0.58	1.36	1.74	1.43	2E-02	1E-02	1E-08	2E-10	4E-07	28.09
Col18a1	-0.04	0.05	0.59	1.43	1.05	9E-01	9E-01	1E-03	2E-10	5E-09	0.65	0.84	1.12	1.26	1.21	3E-05	1E-08	9E-13	6E-13	4E-11	39.33
Col6a3	0.35	0.42	1.15	1.50	1.04	3E-01	2E-01	1E-09	1E-10	9E-09	0.54	0.62	1.02	1.12	0.87	8E-04	3E-05	9E-11	7E-11	6E-07	102.88
Col6a2	0.34	0.57	0.93	1.60	1.20	4E-01	7E-02	2E-06	7E-11	8E-10	0.47	0.95	1.01	0.95	0.61	6E-03	8E-09	1E-09	9E-08	1E-03	91.74
Col6a1	0.32	0.38	0.79	1.37	1.19	3E-01	2E-01	4E-06	3E-10	2E-11	0.52	0.84	0.81	0.87	0.73	6E-04	9E-09	2E-08	4E-08	9E-06	145.66
Col14a1	0.04	0.37	0.82	0.89	0.52	9E-01	8E-02	2E-09	9E-09	2E-05	0.17	0.44	0.69	0.87	0.73	1E-01	2E-05	5E-10	8E-12	4E-08	22.48
Col27a1	-0.13	0.27	0.28	1.05	0.43	7E-01	4E-01	1E-01	6E-08	6E-03	0.23	0.40	0.24	0.35	0.23	1E-01	1E-03	8E-02	3E-02	2E-01	10.42
Col4a5	0.21	0.21	0.84	0.83	0.37	4E-01	4E-01	1E-09	2E-07	3E-03	-0.45	0.07	0.43	0.56	0.19	1E-04	5E-01	6E-05	3E-06	2E-01	44.05
Col4a4	0.24	0.37	0.47	0.59	0.30	5E-01	2E-01	5E-03	2E-03	6E-02	-0.99	-0.38	0.19	0.46	0.15	2E-10	5E-03	2E-01	3E-03	4E-01	38.21
Col4a3	0.78	0.31	0.47	0.47	0.30	6E-02	6E-01	6E-02	1E-01	2E-01	-0.86	-0.60	0.11	0.19	0.08	6E-05	2E-03	6E-01	5E-01	8E-01	91.39
Col10a1	-0.21	-0.04	-0.64	-0.32	-0.79	8E-01	1E+00	2E-02	3E-01	6E-04	-0.52	-0.84	-1.06	0.34	0.18	1E-02	1E-04	8E-06	2E-01	6E-01	3.45
Col13a1	0.40	0.17	0.21	0.10	-0.42	2E-01	7E-01	3E-01	7E-01	2E-02	0.07	-0.09	-0.40	-0.37	-0.69	7E-01	6E-01	5E-03	3E-02	2E-04	22.65
Col4a6	-0.36	-0.02	0.13	-0.11	-0.46	3E-01	1E+00	6E-01	6E-01	6E-03	-0.60	-0.52	-0.68	-0.41	-0.74	2E-04	3E-04	1E-05	3E-02	3E-04	8.80
Col20a1	-0.34	-0.11	-0.41	-0.63	-0.96	4E-01	9E-01	1E-01	2E-02	6E-05	-0.57	-0.41	-0.64	-0.47	-0.68	2E-03	3E-02	2E-03	4E-02	1E-02	3.40
Col17a1	-0.98	-0.52	-1.41	-0.75	-1.10	5E-02	3E-01	8E-06	5E-02	1E-04	-0.75	-1.10	-1.59	-1.89	-1.67	4E-03	1E-06	4E-08	1E-07	2E-05	4.49

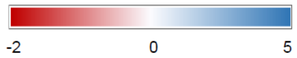

b

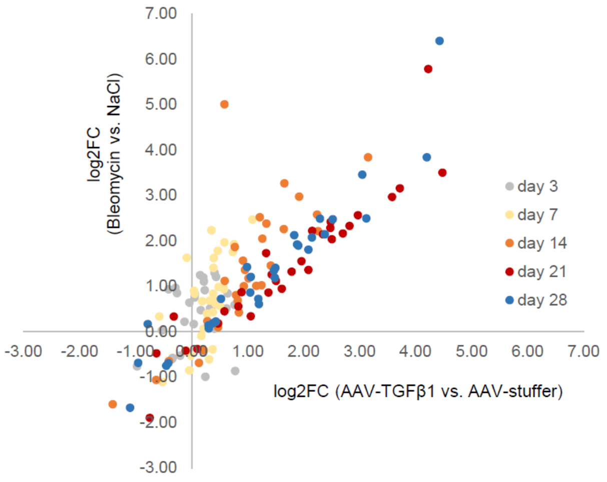

c

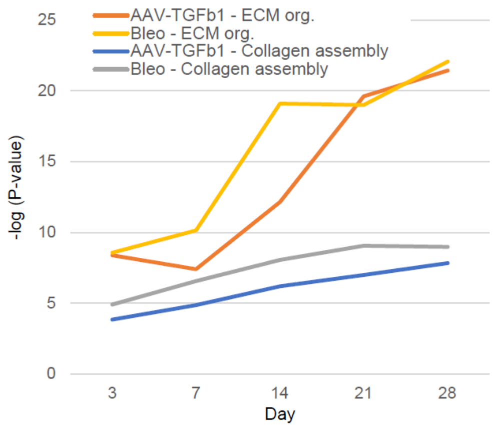

Suppl. Fig. 3: Collagen expression and ECM formation. (**a**) Expression data for all robustly expressed collagens (defined as an FPKM >=1 in at least one contrast/time point) from AAV-TGFβ1 vs. AAV-stuffer and Bleomycin vs. NaCl samples. The data were limited to collagens that showed differential expression (abs. logFC >=0.6, adj. p<0.05) in at least one contrast/time point across models. Yellow indicates adj. p-values<0.05. (**b**) Correlation plot for the log_2FC values from (a), separated by day. (**c**) Enrichment of the Reactome pathways "Extracellular matrix organization Homo sapiens R-HSA-1474244" (ECM org.) and "Assembly of collagen fibrils and other multimeric structures Homo sapiens R-HSA-2022090" (Collagen assembly) over time, depicted as the -log(P-value) of enrichment.

Supplemental Figure 4

Pre-defined patterns for correlation analysis:

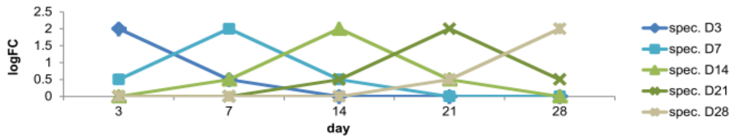

UPREGULATED genes:

		spec. D3			spec. D7			spec. D14			spec. D21			spec. D28		
		Top 10 genes	logFC	Profile	Top 10 genes	logFC	Profile	Top 10 genes	logFC	Profile	Top 10 genes	logFC	Profile	Top 10 genes	logFC	Profile
AAV-TGFβ1		Gm20459	1.31		Grik4	1.62		Slc26a4	3.71		Ighv1-81	4.32		Igkv4-61	3.90	
		Gm20603	0.86		Cd4	0.83		Ighv5-16	3.62		Ighv1-4	4.23		Awat1	3.58	
		Arid3a	0.69		Slamf6	0.81		Exo1	2.48		Wfdc12	4.05		Gm15725	3.55	
		Ndst1	0.57		Blnk	0.69		Pappa2	2.40		Ighv1-34	4.04		Igkv4-74	3.39	
		Tapbpl	0.54		Senp1	0.29		Neil3	2.28		Ucma	3.96		Sectm1a	3.27	
		Bcl7b	0.42		Clint1	0.26		Ighv1-52	2.23		Igkv4-57-1	3.76		Ntsr2	3.25	
		Shisa5	0.37					Pla2g4c	2.08		Ighv1-15	3.75		AU018091	3.23	
		Specc1	0.34					Gen1	1.93		Atf7ip2	3.65		Ighv1-82	3.21	
								Nr5a2	1.93		Bcat1	3.53		Gm807	3.18	
								Gm17300	1.75		Raet1d	3.43		Prss46	3.14	
	survivors, n=	8			6			152			326			186		
	Reactome/	HSPG biosynthetic process	0.006		Signaling by Interleukins	0.005		Cell Cycle	6.3E-08		Pos. reg. of resp. burst	0.009		Platelet activation, aggregat.	0.010	
	GO biol. Process	Pos. reg. of cell cycle arrest	0.033		TH-17 cell lineage commitm	0.002					Reg. of T cell apoptosis	0.001		ECM organization	0.017	
	enrichment															
	ARCHS^4 kinase	---			SCYL2 kinase	6.4E-05		CDK1 kinase	8.0E-17		DAPK3 kinase	1.9E-15		---		
	co-expression							PLK4 kinase	8.0E-17		MAP3K6 kinase	6.5E-13				
Bleomycin		Tgtp1	3.02		Sectm1b	3.89		Ereg	5.52		Ighv1-76	3.40		Ighv7-3	4.31	
		Irf7	2.88		Cpa4	3.47		Gjb4	4.19		H2-M2	3.34		Iglv1	4.30	
		Lcn2	2.79		R3hdml	3.37		Iqgap3	3.61		Ighv1-54	3.20		Igkv5-48	4.28	
		Mir546	2.73		Cd209e	3.07		A2m	3.56		Ighv9-4	3.05		Ighv14-3	3.84	
		Mx1	2.61		Chit1	3.06		Nog	3.55		Ksr2	2.88		Igkv3-12	3.73	
		Ifi44	2.38		Cdca5	3.05		Pdcd1	3.55		Cpz	2.87		Iglv2	3.71	
		Oas1a	2.32		Hmmr	2.94		AU018091	3.29		Ighv13-2	2.84		Ighv1-26	3.58	
		Zbp1	2.26		Hnf4a	2.94		Clca1	3.26		Igkv6-14	2.81		Cldn2	3.45	
		Ifi27l2a	2.24		Cdca8	2.93		Acan	2.85		Ighv1-63	2.59		Igkv19-93	3.42	
		Ifit1	2.23		Ccl17	2.77		Btbd17	2.84		Rgn	2.44		Gm16710	3.42	
	survivors, n=	336			173			124			88			95		
	Reactome/	Interferon Signaling	2.7E-20		Cell Cycle	1.1E-07		Signaling by B Cell Recept.	0.015		COPII Vesicle Transport	0.001		Signaling by NOTCH3	0.001	
	GO biol. Process	Cytokine Signaling in	2.5E-11		Ox. Stress Induced Senesc.	1.4E-05		Fibrin Clot Formation	0.008		PDGFR signaling pathway	0.006		Neg. reg. of osteoblast diff.	3.1E-04	
	enrichment	Immune Sytem			Infectious disease	1.1E-05		Reg. of cytokine biosynth.	0.003							
	ARCHS^4 kinase	EIF2AK2 kinase	1.8E-23		SRPK1 kinase	1.5E-11		VRK2 kinase	0.001		ACVR1 kinase	0.001		---		
	co-expression	RIPK1 kinase	1.2E-18		PLK1 kinase	1.5E-11					PDGFRA kinase	0.001				

DOWNREGULATED genes:

		spec. D3			spec. D7			spec. D14			spec. D21			spec. D28		
		Top 10 genes	logFC	Profile	Top 10 genes	logFC	Profile	Top 10 genes	logFC	Profile	Top 10 genes	logFC	Profile	Top 10 genes	logFC	Profile
AAV-TGFβ1		Kcna6	-1.81		Wrap73	-0.52		Gm15564	-5.75		Fabp1	-5.18		Gm23973	-3.91	
		Ngef	-0.78		Zfand2b	-0.48		Gm17035	-3.77		Atp2b2	-3.64		1700123L14Rik	-3.67	
		Slc26a2	-0.59		Sh3bp5l	-0.40		Gm11175	-3.12		9230117E06Ri	-3.61		Klra9	-3.20	
		Idh2	-0.44					2300005B03Ri	-2.99		Crb1	-3.58		Gm26736	-3.09	
								Bai1	-2.96		Gm12725	-3.54		Gm24530	-3.06	
								En1	-2.82		Cyp4a32	-3.53		Klra7	-3.02	
								Cilp2	-2.78		Gm7741	-3.35		Gm15902	-2.76	
								Pou3f3	-2.76		4930471I20Rik	-3.33		Gm13314	-2.69	
								Sdr9c7	-2.76		Myh7	-3.11		Gm12014	-2.66	
								Gm25238	-2.75		Snord32a	-3.06		Gm15509	-2.63	
	survivors, n=	4			3			245			323			326		
	Reactome/	---			Pos. reg. of cilium assembly	0.003		reg. of transcription	2.0E-06		PI-3K cascade:FGFR1-4	0.004		---		
	GO biol. Process							epithelial cell development	0.001							
	enrichment															
	ARCHS^4 kinase	---			---			TNK2 kinase	4.6E-06		---			---		
	co-expression							MAP3K6 kinase	4.6E-06							
Bleomycin		A530058N18Ri	-2.73		Igfbp3	-3.01		4930551L18Ri	-2.76		Amer2	-3.14		Cyp1a1	-4.85	
		Gm15857	-2.08		Cdh16	-2.23		Gm16102	-2.49		Foxd4	-3.11		Slc7a10	-3.96	
		Cdc73	-1.91		1110035H17Ri	-2.04		Pnpla5	-2.37		Lin28a	-2.42		Gsta2	-2.68	
		Gm11670	-1.86		Gm12741	-1.98		Gm15883	-2.28		Gm12983	-2.42		9530036M11Ri	-2.62	
		A730056A06Ri	-1.48		4930564G21Ri	-1.89		9330171B17Ri	-2.22		Spatc1	-2.38		Olfr750	-2.58	
		Gm8425	-1.43		Rps6-ps1	-1.69		Gm24173	-2.20		Gm13570	-2.38		Cyp2g1	-2.47	
		Mir3074-1	-1.36		Rs1	-1.65		Ccdc129	-2.02		Gm16331	-2.38		Gm2830	-2.29	
		C430014B12Ri	-1.29		Gm10532	-1.61		Gm21817	-2.00		Scrg1	-2.36		Glra4	-2.27	
		Gbx1	-1.22		Insm2	-1.51		1700034I23Rik	-1.97		Gad1-ps	-2.36		Sypl2	-2.11	
		Gm26664	-1.17		Gm11511	-1.50		Gm11447	-1.97		Capn13	-2.35		Gm11343	-2.10	
	survivors, n=	152			233			195			148			130		
	Reactome/	Signaling by Wnt	0.007		RORA activates gene expr.	1.3E-05		---			---			Signal. by WNT in cancer	0.001	
	GO biol. Process				Chromatin modif. Enzymes	7.3E-05								Interleukin-1 signaling	0.033	
	enrichment															
	ARCHS^4 kinase	IGF1R kinase	9.9E-05		TAOK1 kinase	1.6E-16		---			SPEG kinase	3.6E-07		---		
	co-expression	MAP4K4 kinase	9.9E-05		BRAF kinase	1.5E-15					ALPK3 kinase	3.6E-07				

Suppl. Fig. 4: Expression correlation to pre-define patterns. Temporal gene expression patterns were defined (see line graph) to identify genes with peak expression levels at respective time points. Strongly correlating (Pearson r ≥|0.9|) genes that showed significant alteration (adj. p ≤0.05) were selected ("survivors") and ranked by their log_2-fold change at the respective time point of interest. Expression profiles over all time points are displayed as bar graphs for each gene. Surviving genes were further applied to pathway enrichment analysis to identify associated processes, the top 3 of which are shown (if applicable) along with p-values describing the significance of enrichment.

Supplemental Figure 5

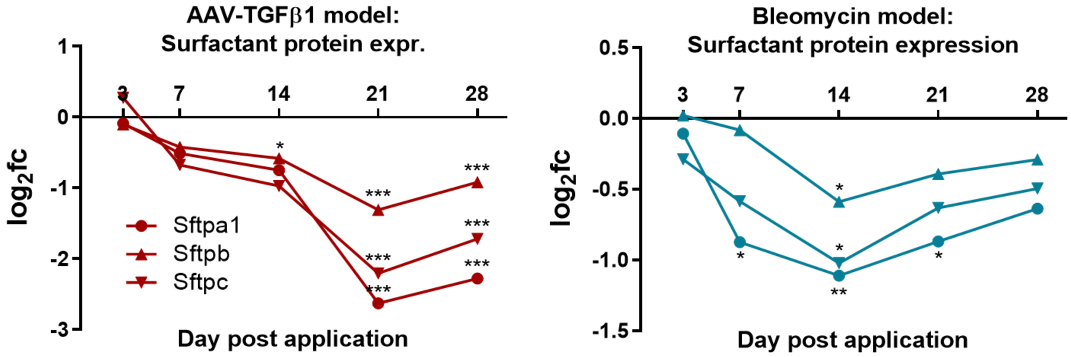

Suppl. Fig. 5: Expression of Surfactant proteins. RNA-seq derived mean gene expression values of Surfactant proteins A, B and C over time in both models. *adj. p<0.05, **adj. p<0.01, ***adj. p<0.001.

Supplemental Figure 6

#	Pathway (GO Biological Process)	Bleomycin p	Bleomycin adj. p	AAV-TGFb1 p	AAV-TGFb1 adj. p	Δ adjP	Pathway genes altered in each model: AAV-TGFb1	Pathway genes altered in each model: Bleomycin
1	cellular response to **type I interferon** (GO:0071357)	0.0000	0.0032	0.9246	1.0000	0.997	ISG20;SP100;RSAD2;MX2;IRF5;GBP2;XAF1;MYD88	**IFITM3;IFITM1;IFITM2;RSAD2;STAT1;MX2;STAT2;MX1;ADAR;ISG15;IFI35;IFIT1;IFIT3;PSMB8;IFIT2;BST2;IFI27;OAS2;IRF7;IRF5;GBP2;XAF1;IRF9;MYD88**
2	negative regulation of **leukocyte activation** (GO:0002695)	0.0037	0.0727	0.6220	1.0000	0.927	FCGR2B;HAVCR2	**MILR1;CD84;CD300A;FCGR2B;CD300LF;HAVCR2**
3	positive regulation of **innate immune response** (GO:0045089)	0.0046	0.0842	0.5522	0.9982	0.914	CCL5;WNT5A;ADAM8;LBP;DDX60;TLR5;TLR2	**ZBP1;GBP5;WNT5A;NLRC5;DDX60;CCL5;DHX58;TLR9;TLR8;ADAM8;LBP;TLR5;TLR2**
4	positive regulation of **tumor necrosis factor** production (GO:0032760)	0.0001	0.0046	0.3477	0.8336	0.829	PYCARD;CD2;WNT5A;LBP;CD14;MIF;THBS1;CLU;HAVCR2;TLR2	**DDX58;WNT5A;LY96;MIF;THBS1;CLU;RASGRP1;IFIH1;PYCARD;CD2;TLR1;TLR9;LBP;CD14;CCL19;LGALS9;CCR2;HAVCR2;TLR2**
5	positive regulation of T cell activation (GO:0050870)	0.0006	0.0246	0.3514	0.8336	0.809	CD86;ANXA1;VCAM1;TFRC;GATA3;AIF1;PYCARD;PTPRC;IL1B;CCL5;SIRPA;PRKCQ;CD46;JAK3	**CD86;FAM49B;CD274;XBP1;ANXA1;VCAM1;THY1;GATA3;AIF1;PYCARD;CCDC88B;PNP;IL7;IL1B;CCL5;SIRPA;NCKAP1L;LGALS9;CCL19;CD46;JAK3;CCR2**
6	response to interferon-gamma (GO:0034341)	0.0006	0.0246	0.3514	0.8336	0.809	GBP6;SP100;CCL11;CCL22;SLC11A1;WNT5A;AQP4;AIF1;CXCL16;CCL8;CCL5;CCL17;SLC26A6;SNCA	**IFITM3;GBP6;IFITM1;IFITM2;CCL11;CCL22;GCH1;STAT1;SLC11A1;WNT5A;AIF1;CX3CL1;CXCL16;BST2;CCL8;KYNU;CCL5;LGALS9;CCL19;CCL17;SLC26A6;SNCA**
7	positive regulation of **interleukin-6** production (GO:0032755)	0.0019	0.0494	0.3804	0.8391	0.790	PYCARD;IL1B;F2R;WNT5A;F2RL1;LBP;MYD88;MAPK13;TLR2	**XBP1;DDX58;F2R;WNT5A;MAPK13;IFIH1;PYCARD;TLR1;IL1B;TLR9;TLR8;LBP;LGALS9;MYD88;TLR2**
8	regulation of interleukin-10 secretion (GO:2001179)	0.0005	0.0226	0.3006	0.7955	0.773	PYCARD;PRKCZ	**PYCARD;CD274;LGALS9;PRKCZ;TNFRSF21**
9	proteolysis involved in cellular protein catabolic process (GO:0051603)	0.0024	0.0575	0.3279	0.8210	0.763	CTSA;GZMA;CTSZ;ADAMTS12;CTSS;HDAC6;PSMA5;SCPEP1;PSMB7;PSMB5;FAP;CTSL;CTSK;CTSH;PMAIP1;CTSF;CTSC;CTSB;LGMN	**CTSZ;TNFAIP3;ADAMTS12;CTSS;PSMB10;HDAC6;PSMB6;SCPEP1;PSMB5;CTSL;CTSK;CTSH;CTSF;TINAG;CTSC;CTSB;CTSA;GZMA;ISG15;NR1D1;PSMB8;PSMA5;PSMA4;FAP;UBA52;LGMN**
10	antigen processing and presentation … via MHC class I (GO:0042590)	0.0044	0.0822	0.3415	0.8336	0.751	PDIA3;PSMD10;FCER1G;NCF1;ITGB5;PSMD14;PSMD13;IFI30;PSMD8;PSMA5;SEC14L3;PSMB7;PSMB5;PSMD2;ITGAV;CALR	**FCER1G;NCF1;ITGB5;PSMD14;NCF2;NCF4;TAP1;CYBB;IFI30;PSMB10;PSMB8;PSMD8;PSMA5;PSMB6;SEC14L3;PSMA4;PSMB5;PSME1;PSME2;ITGAV;CALR;B2M**
1	protein insert. into mitochond. membrane … **apoptotic signal. pathway** (GO:0001844)	0.6486	0.9942	0.0000	0.0041	0.990	**MOAP1;BCL2L11;BAD;BAX;PMAIP1;BMF**	MOAP1
2	mitochondrial outer membrane permeabilization (GO:0097345)	0.5944	0.9942	0.0003	0.0200	0.974	**MOAP1;BCL2L11;BAD;BLOC1S2;BNIP3;BAX;PMAIP1;BMF**	MOAP1;BLOC1S2
3	positive regulation of **cellular response to TGF-beta stimulus** (GO:1903846)	0.3617	0.7907	0.0034	0.0921	0.699	**CDKN1C;SDCBP;FLCN;CDKN2B;DAB2;CITED2;GIPC1;THBS1;HIPK2**	SDCBP;DAB2;CITED2;THBS1
4	**lung development** (GO:0030324)	0.3147	0.7283	0.0031	0.0865	0.642	**ERRFI1;CEBPA;CCDC39;WNT11;PDPN;LOXL3;NKX2-1;TCF21;CHI3L1;FGFR2;VEGFA;FGF10**	PDPN;LOXL3;NKX2-1;TCF21;FGFR2;VEGFA
5	pos. regulation of cardiac muscle contract (GO:0060452)	0.2471	0.6672	0.0010	0.0418	0.625	**RGS2;KCNQ1;NPPA;ADRA1A;TRPM4**	NPPA;ADRA1A
6	**glutathione** derivative biosynthetic process (GO:1901687)	0.2692	0.6982	0.0030	0.0849	0.613	**GSTM4;GSTZ1;GSTK1;GSTM1;GSTA4;GSTA3;ESD;MGST1;MGST2;GSTM5**	GSTM1;GSTO1;GSTA3;ESD;MGST2
7	negative regulation of protein processing (GO:0010955)	0.2090	0.6355	0.0004	0.0225	0.613	**PRNP;SERPINE2;LRRK2;SERPINE1;CTSZ;GAS1;CHAC1;THBS1;CD55**	SERPINE2;SERPINE1;CTSZ;THBS1
8	**epithelial cell morphogenesis** (GO:0003382)	0.2062	0.6355	0.0005	0.0253	0.610	**CLDN3;RILPL2;RAB25;POF1B;MET;SIPA1L3;GRHL2**	RILPL2;POF1B;MET
9	peptide metabolic process (GO:0006518)	0.2174	0.6557	0.0026	0.0803	0.575	**CPM;ENPEP;CPXM2;PROS1;SPPL2B;MGST1;FURIN;AEBP1;PCSK5;DNPEP;ANPEP;CPXM1;DMD;SEC11A;GSTM4;GSTK1;HSP90AA1;ACE;GSTM1;MME;F10;EEF1G;GSTZ1;F7;ALDH5A1;SPCS1;GSTA4;GSTA3;GAS6;PTGES;GSTM5**	CPM;ENPEP;GSTM1;MME;F10;CPXM2;GSTO1;PROS1;FURIN;AEBP1;PCSK5;F7;IMMP2L;ALDH5A1;DNPEP;GSTA3;CPXM1;DMD;EEF1E1;PTGES
10	epithelial cell development (GO:0002064)	0.2458	0.6672	0.0038	0.0948	0.572	**CLDN3;RILPL2;RAB25;POF1B;WNT7A;SIPA1L3;GRHL2;EXPH5**	RILPL2;POF1B;TMEM79;EXPH5

Suppl. Fig. 6: Differentially activated pathways and genes. Shown are the top 10 pathways preferentially enriched in the respective model, defined and ranked by the difference in pathway enrichment as described in detail in main Figure 3. In addition to Fig. 3, this figure depicts all individual genes altered in the enriched pathways.

Supplemental Figure 7

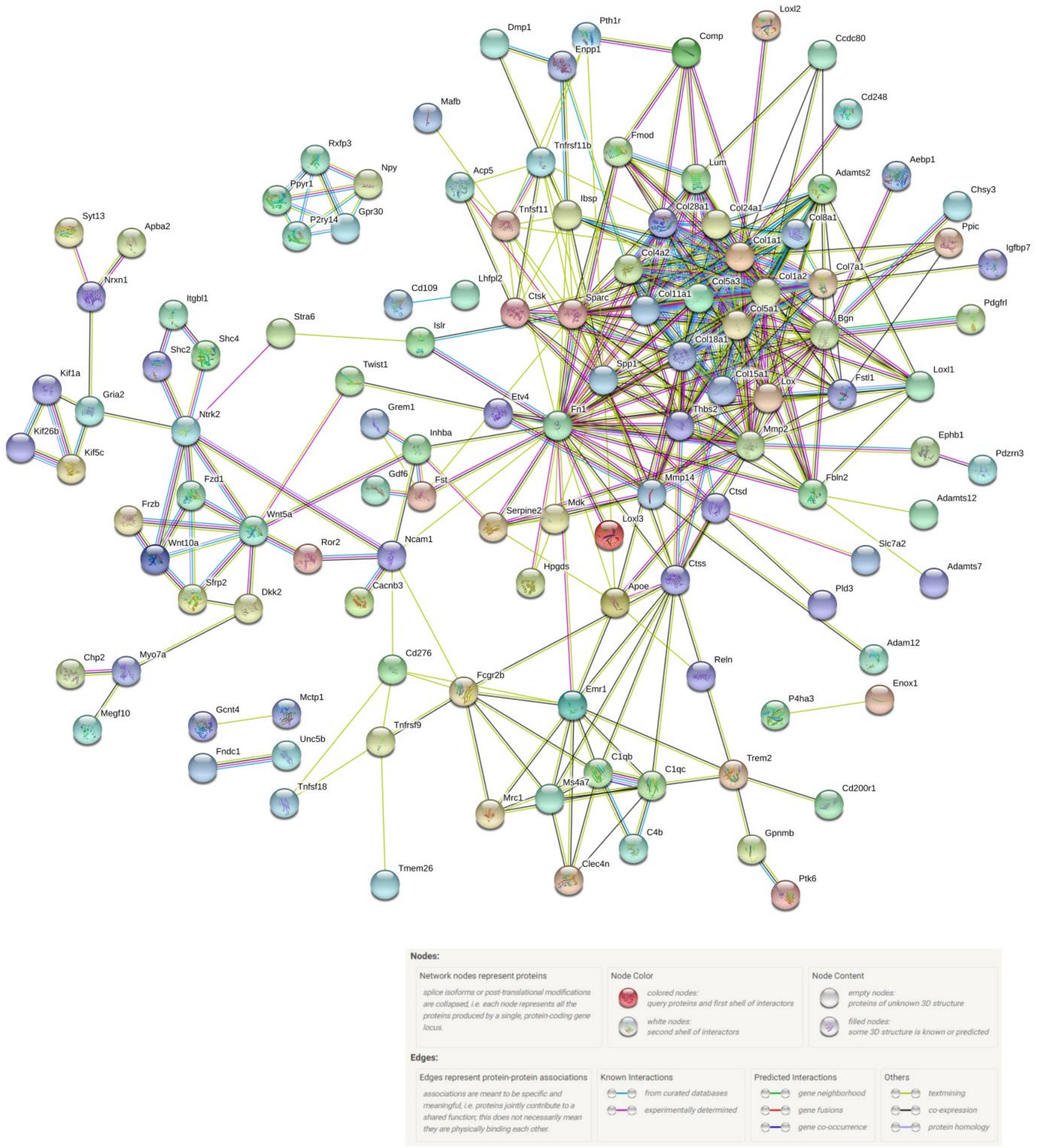

Suppl. Fig. 7: STRING protein-protein interaction network for the top 200 upregulated genes whose expression was anti-correlated with lung function, as detailed in Fig. 4. Image created using STRING (https://string-db.org/) version 10.5.

Supplemental Figure 8

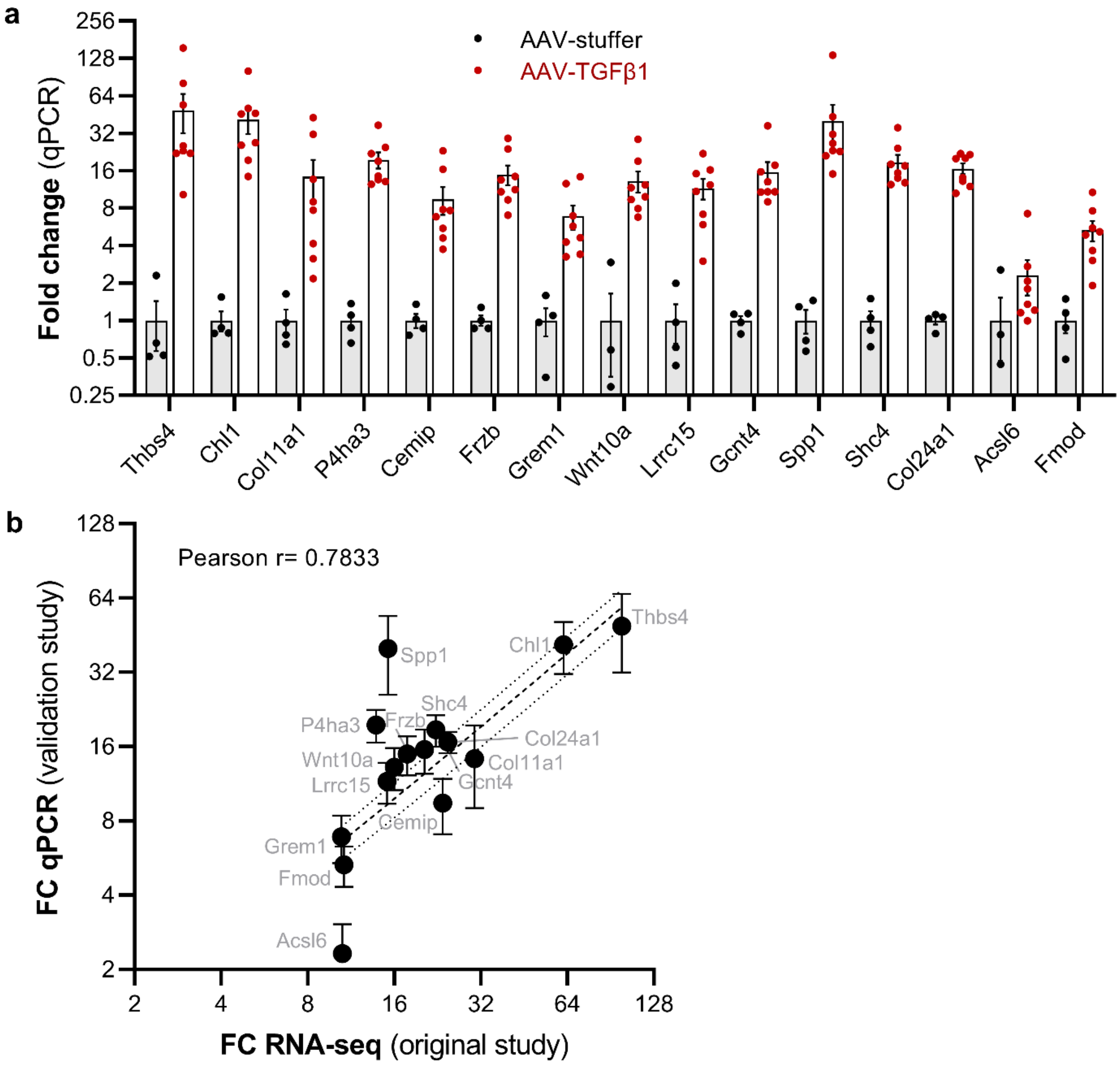

Suppl. Fig. 8: Validation of the top-15 commonly upregulated mRNAs by qPCR. To technically and biologically validate the expression of the commonly upregulated, lung-function anti-correlated genes on day 21 (compare main Fig. 4a), the expression of the top-15 genes was assessed in a follow-up validation study via qPCR. Mice again received 2.5E+11 VG AAV-stuffer or AAV-TGFβ1 by intratracheal administration and gene expression was assessed 21 days later in total lung RNA samples. (**a**) Expression of the depicted genes, measured via qPCR, expressed as the fold change in expression in AAV-TGFβ1 treated animals, compared to AAV-stuffer. n= 4 (AAV-stuffer) and n=8 (AAV-TGFβ1), Mean±SEM. (**b**) Correlation of the gene expression changes (FC, fold change) measured by RNA-seq in the original study and by qPCR in the validation study. The dashed line represents a linear regression with 90% confidence intervals. Mean±SEM. Log_2-scaled axes are used in both (a) and (b) to allow assessment of comparatively small values.

Supplemental Figure 9

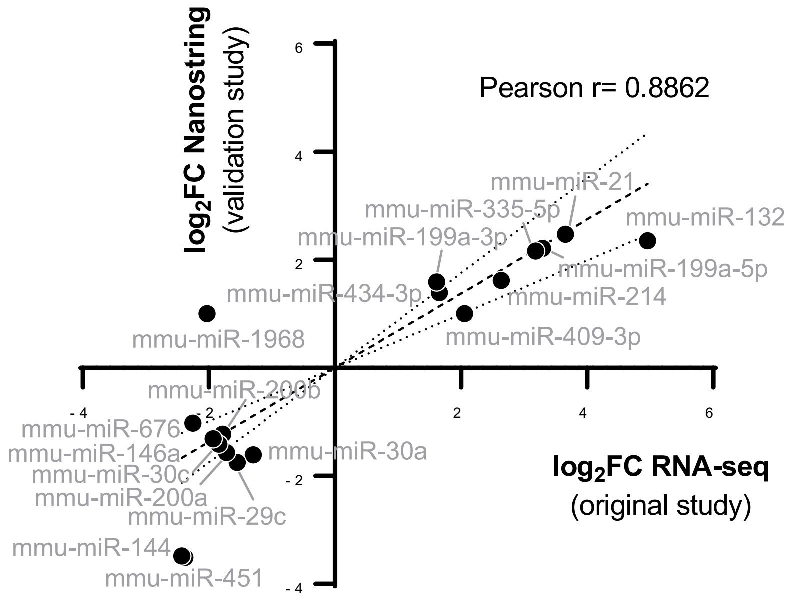

Suppl. Fig. 9: Validation of miRNA expression by Nanostring nCounter analysis. To technically and biologically validate the expression of the commonly deregulated, lung-function (anti-)correlated miRNAs on day 21 (as shown in main Fig. 5c), the expression of these miRNAs was assessed in a follow-up validation study (compare Suppl. Fig. 8) via Nanostring nCounter analysis. Expression changes between AAV-TGFβ1 treated animals and AAV-stuffer were analyzed using Nanostring nSolver software (settings: background threshold 50, normalization over top 100) and expressed as mean log2-fold changes. n= 4 (AAV-stuffer) and n=8 (AAV-TGFβ1). A correlation plot for the gene expression changes measured by RNA-seq in the original study and by Nanostring nCounter analysis in the validation study, is shown. The dashed line represents a linear regression with 90% confidence intervals.

Supplemental Figure 10

miRNA	extracellular.matrix.organization	regulation.of.fibroblast.proliferation	positive.regulation.of.fibroblast.proliferation	negative.regulation.of.fibroblast.proliferation	Inflammation_Complement.system	Cell.adhesion_Cell.matrix.interactions	Pulmonary.Fibrosis	Idiopathic.Pulmonary.Fibrosis	Hypoxia.response.regulation	Immune.system.response	Tissue.remodeling.and.wound.repair
mmu-miR-148a-3p	0	0	0	0	0	0	0	0	0	0	1,2
mmu-miR-148b-3p	0,5	0	0	0	0	0,3	0	0	0	0	0
mmu-miR-181a-5p	4,6	0	0,5	0	0	1	0,9	0,9	0	3	4,2
mmu-miR-10a-5p	0	0,2	0,4	0	1,9	0	3,2	2,1	0	0	0
mmu-miR-181b-5p	3,6	0	0	0	0	0,5	0	0	1	1,3	2
mmu-miR-652-3p	0	1,8	0	2,5	0	0	2,1	2,4	0	0	0
mmu-miR-151-3p	0	0	0	0	0	0	0	0,1	0	0	0
mmu-miR-195a-5p	0	0	0	0	0	0	0	0,2	0	0,9	0
mmu-miR-503-3p	0	0	0	0	0	0	0	0	0	0	0,4
mmu-miR-203-3p	1	0	0	0	0	0	0	0	0	0,9	1,3

Suppl. Fig. 10: Pathway enrichment of predicted miRNA target sets. Predicted target sets for each miRNA were applied to metabase pathway enrichment analysis. The table shows -log(adj.-p) values for a subset of downregulated miRNAs. Higher values indicate stronger enrichment.
